# A purine metabolic checkpoint that prevents autoimmunity and autoinflammation

**DOI:** 10.1016/j.cmet.2021.12.009

**Published:** 2022-01-04

**Authors:** Svetlana Saveljeva, Gavin W. Sewell, Katharina Ramshorn, M. Zaeem Cader, James A. West, Simon Clare, Lea-Maxie Haag, Rodrigo Pereira de Almeida Rodrigues, Lukas W. Unger, Ana Belén Iglesias-Romero, Lorraine M. Holland, Christophe Bourges, Muhammad N. Md-Ibrahim, James O. Jones, Richard S. Blumberg, James C. Lee, Nicole C. Kaneider, Trevor D. Lawley, Allan Bradley, Gordon Dougan, Arthur Kaser

**Affiliations:** 1 Cambridge Institute of Therapeutic Immunology and Infectious Disease, Jeffrey Cheah Biomedical Centre, University of Cambridge, Cambridge CB2 0AW, UK; 2 Division of Gastroenterology and Hepatology, Department of Medicine, University of Cambridge, Addenbrooke’s Hospital, Cambridge CB2 0QQ, UK; 3 Wellcome Trust Sanger Institute, Hinxton, Cambridge CB10 1SA, UK; 4 Division of Gastroenterology, Hepatology and Endoscopy, Department of Medicine, Brigham and Women’s Hospital and Harvard Medical School, Boston, MA 02115, USA; 5 Division of Infectious Diseases, Department of Medicine, University of Cambridge, Cambridge CB2 0QQ, UK

**Keywords:** autoimmunity, dendritic cells, T cell priming, purine nucleotide cycle, NADH/NAD^+^ reductive stress, membrane trafficking

## Abstract

Still’s disease, the paradigm of autoinflammation-*cum*-autoimmunity, predisposes for a cytokine storm with excessive T lymphocyte activation upon viral infection. Loss of function of the purine nucleoside enzyme FAMIN is the sole known cause for monogenic Still’s disease. Here we discovered that a FAMIN-enabled purine metabolon in dendritic cells (DCs) restrains CD4^+^ and CD8^+^ T cell priming. DCs with absent FAMIN activity prime for enhanced antigen-specific cytotoxicity, IFNγ secretion, and T cell expansion, resulting in excessive influenza A virus-specific responses. Enhanced priming is already manifest with hypomorphic FAMIN-I254V, for which ∼6% of mankind is homozygous. FAMIN controls membrane trafficking and restrains antigen presentation in an NADH/NAD^+^-dependent manner by balancing flux through adenine-guanine nucleotide interconversion cycles. FAMIN additionally converts hypoxanthine into inosine, which DCs release to dampen T cell activation. Compromised FAMIN consequently enhances immunosurveillance of syngeneic tumors. FAMIN is a biochemical checkpoint that protects against excessive antiviral T cell responses, autoimmunity, and autoinflammation.

## Highlights

• A FAMIN-enabled purine metabolon in dendritic cells restrains T cell priming
• FAMIN prevents cytoplasmic NADH/NAD^+^ reductive stress that enhances priming
• Inosine generated from hypoxanthine by FAMIN inhibits T cell priming
• FAMIN ameliorates immunopathology in influenza but dampens tumor surveillance

## Graphical abstract

**Figure:**
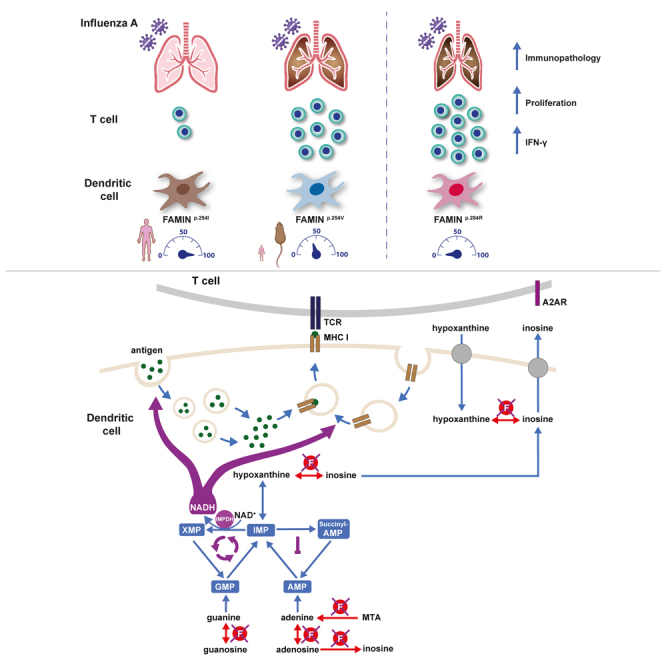

## Teaser

Saveljeva et al. identify a biochemical mechanism in dendritic cells that restrains T cell priming and prevents immunopathology but dampens tumor surveillance. FAMIN enables a purine nucleotide cycle, which prevents cytoplasmic NADH/NAD^+^ reductive stress that augments antigen presentation, and it generates inosine, which inhibits T cell activation.

## Introduction

Deorphaning an autoimmunity risk gene product unearthed an unprecedented function at the heart of cellular metabolism, conserved from bacteria to man. This risk gene encodes FAMIN (also known as LACC1, C13orf31), an enzyme that unifies in a single protein the activities of adenosine deaminase (ADA; adenosine + H_2_O ⇀ inosine + NH_3_), purine nucleoside phosphorylase (PNP; inosine + phosphate [P_i_] ⇌ hypoxanthine + ribose-1-phosphate [R1P]; guanosine + P_i_ ⇌ guanine + R1P), and methylthioadenosine phosphorylase (MTAP; methylthioadenosine [MTA] + P_i_ ⇌ adenine + methyl-thioribose-1-phosphate [MTR1P]). FAMIN’s fourth catalytic activity is that of an adenosine phosphorylase (adenosine + P_i_ ⇌ adenine + R1P), previously considered absent from eukaryotic metabolism (Cader et al., 2020). Adenine and ribose are primordial metabolites from which life is thought to have emerged from prebiotic biochemistry (Ralser, 2018). They are defining constituents of the genetic code, the energy currency, and the major cofactors of a cell. Since purine nucleotide *de novo* synthesis yields straight to nucleotides (i.e., purine monophosphates; IMP, AMP, and GMP), ADA, PNP, and MTAP had been thought to be the sole enzymes to supply purine nucleobases (adenine, guanine, and hypoxanthine) from nucleosides (adenosine, guanosine, inosine, and MTA) (Bzowska et al., 2000). ADA and PNP deficiency causes severe combined immunodeficiency (SCID) with loss of T and B lymphocytes (Giblett et al., 1972, 1975). Loss of function of FAMIN, in sharp contrast, is linked to autoimmunity and autoinflammation, specifically to Still’s disease (also known as systemic juvenile idiopathic arthritis, sJIA), juvenile idiopathic arthritis (JIA), and early-onset Crohn’s disease (Al-Mayouf et al., 2020; Patel et al., 2014; Rabionet et al., 2019; Wakil et al., 2015; Yasin and Schulert, 2018). These very rare loss-of-function mutations aside, partial loss of activity, caused by a SNP that leads to a valine-for-isoleucine substitution at amino acid 254 (I254V), for which ∼6% of humans are homozygous, increases risk of Crohn’s disease and leprosy (Barrett et al., 2008; Zhang et al., 2009).

FAMIN loss of function is the sole known cause of autosomal-recessive (i.e., monogenic) forms of Still’s disease. Still’s disease affects young children; starts with daily recurring fever, rash, and lymph node enlargement; and morphs over weeks into a debilitating arthritis (Yasin and Schulert, 2018). The initial phase resembles periodic fever syndromes with inflammasome activation and IL-1β and IL-18 release; the later arthritic phase is thought to be driven by pathogenic T lymphocytes. About 20% of children with Still’s disease develop “secondary hemophagocytic lymphohistiocytosis” (HLH; also known as “macrophage activation syndrome,” MAS) (Brisse et al., 2016b; Grom et al., 2016). HLH/MAS is typically triggered by viral infections and can occur even when Still’s disease is in remission while on IL-1/IL-6-blocking therapeutics (Grom et al., 2016). HLH features a cytokine storm accompanied by excessive expansion and activation of CD4^+^ and CD8^+^ T lymphocytes and hemophagocytic, IFNγ-activated macrophages. It manifests with disseminated intravascular coagulation (DIC), acute respiratory distress syndrome, and multi-organ failure, and is often fatal (Brisse et al., 2016a, 2016b). HLH/MAS is not restricted to Still’s disease and children. For example, virus-induced HLH/MAS is caused by Epstein-Barr virus and many other pathogens and has been implicated in fatality from seasonal (H3N2), avian (H5N1), and swine (H1N1/2009) influenza A virus (IAV) infections (Beutel et al., 2011; Henter et al., 2010).

Mice with germline deletion of *Famin*, or genome-edited to express one of the Still’s disease-linked loss-of-function mutations (C284R; “*Famin*^p.284R^” mice), develop normally under specific pathogen-free conditions. Similarly, mice genome-edited to express fully active (254I; “*Famin*^p.254I^”) or partially active FAMIN (254V; “*Famin*^p.254V^”) are indistinguishable (Cader et al., 2016). *Famin*^−/−^ and *Famin*^p.284R^ mice, however, do develop more severe lipopolysaccharide (LPS)-induced sepsis, evidence of DIC, and increased plasma IL-1β levels, compared to mice expressing fully active FAMIN (Cader et al., 2016). Compromised FAMIN activity also leads to lower reactive oxygen species (ROS) production, decreased bacterial killing, altered NLRP3 inflammasome activation, and cytokine secretion in macrophages (Cader et al., 2016; Lahiri et al., 2017), and *Famin*^−/−^ mice develop more severe experimental arthritis and colitis (Kang et al., 2020; Skon-Hegg et al., 2019). How loss of FAMIN activity, which is abundantly expressed in macrophages and dendritic cells (DCs) while largely absent from T cells (Heng et al., 2008), predisposes for autoimmunity remains unknown. Particularly elusive is via what mechanism altered core purine metabolism due to the absence of multifunctional FAMIN, which is tethered to the cytoplasmic surface of peroxisomes (Cader et al., 2016), could affect immune function, since monofunctional ADA, PNP, and MTAP are ubiquitously present.

Here we report a purine metabolon in DCs that potently restrains T cell priming by dampening membrane trafficking and hence the pace of antigen uptake and presentation, and by releasing inosine that provides an inhibitory signal via the adenosine A_2A_ receptor (A_2A_R). Impaired FAMIN catalysis results in excessive IAV-specific T cell responses and lung immunopathology, but also in enhanced tumor immune surveillance. We describe a purely biochemical mechanism within DCs that exerts fundamental control over T lymphocyte priming.

## Results

### FAMIN activity in DCs restrains the influenza A virus-specific T cell response

**Figure 1:**
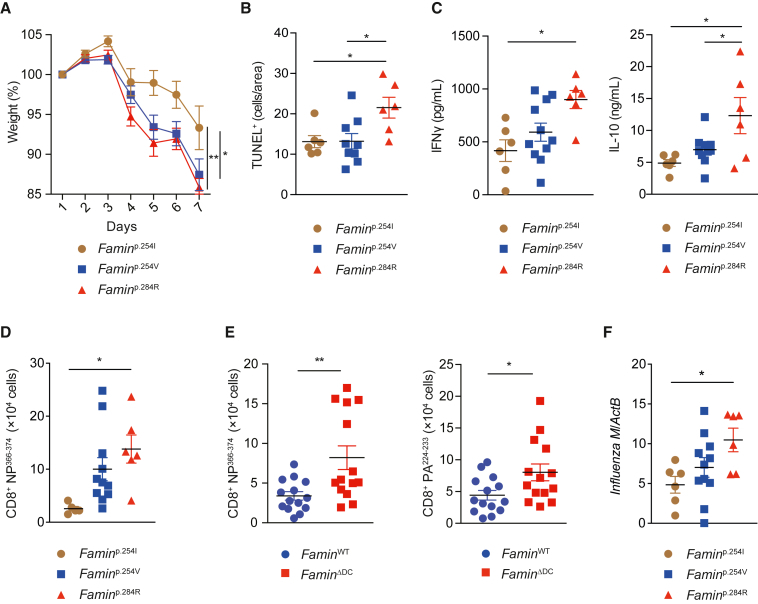
FAMIN deficiency augments T cell responses to influenza A virus (A) Percentage weight loss of *Famin*^p.254I^, *Famin*^p.254V^, *Famin*^p.284R^ mice, which are engineered to express fully active, hypomorphic, and inactive FAMIN, respectively, following infection with influenza A virus (IAV) H3N2, strain A/X-31 (n = 6/11/6). (B) Average number of TUNEL^+^ cells in lungs of *Famin*^p.254I^, *Famin*^p.254V^, and *Famin*^p.284R^ mice 7 days post-infection (n = 6/9/6). (C) Plasma IFNγ and IL-10 levels 7 days after IAV infection (n = 6/11/6). Note IL-12p70 was below the detection limit. (D and E) Absolute numbers of IAV NP^366-374^ tetramer^+^ CD8^+^ T cells in *Famin*^p.254I^, *Famin*^p.254V^, and *Famin*^p.284R^ (D), or CD3^+^CD8^+^NP^366-374^ and PA^224-233^ tetramer^+^ cells in *Famin*^WT^ and *Famin*^ΔDC^ (E) bronchoalveolar lavage fluid (BAL) 7 days after infection (n = 6/11/6; 15/14). (F) IAV M protein gene expression in lung tissue of *Famin*^p.254I^, *Famin*^p.254V^, and *Famin*^p.284R^ mice 7 days after infection (n = 6/11/6). Data represented as mean ± SEM. ^∗^p < 0.05, ^∗∗^p < 0.01, and ^∗∗∗^p < 0.001 (repeated-measures one-way ANOVA, one-way ANOVA, or unpaired two-tailed Student’s t test where appropriate). See also Figure S1.

Originally aiming to gain clues into HLH predisposition, we infected *Famin* mutant mice with a murine-adapted H3N2 IAV strain (A/X-31) (Everitt et al., 2012). *Famin*^p.254V^ and *Famin*^p.284R^ mice, which endogenously express disease-linked hypomorphic and loss-of-function variants, respectively, developed more severe disease compared to *Famin*^p.254I^ mice, which express fully active FAMIN (Figures 1A and S1A). This was associated with more apoptosis, reflecting lung damage (Figure 1B), and elevated plasma IFNγ and IL-10 levels (Figure 1C). Anti-inflammatory IL-10 is produced by IFNγ^+^ IAV-specific CD8^+^ T cytotoxic type 1 cells (Tc1) in IAV-infected lungs (Sun et al., 2009), prompting us to quantify CD8^+^ T cells specific for NP^366-374^, an immunodominant IAV nucleoprotein epitope. On day 7 of infection, numbers of NP^366-374^-reactive CD8^+^ T cells in bronchoalveolar lavage (BAL) were 4-fold higher even in *Famin*^p.254V^ compared to *Famin*^p.254I^, and higher still in *Famin*^p.284R^ mice (Figures 1D and S1B). Cytokine production by Tc1 cells is dependent on their interaction with, and costimulation by, CD11c^+^ DCs infiltrating the infected lung (Hufford et al., 2011). Deletion of *Famin* solely in CD11c^+^ DCs (*Famin*^flox/flox^;*CD11c*-Cre; “*Famin*^ΔDC^”) (Figure S1C) increased the numbers of NP^366-374^-specific and PA^224-233^ (an IAV polymerase acidic protein epitope)-specific CD8^+^ T cells in BAL compared to littermate *Famin*^flox/flox^ mice (“*Famin*^WT^”; Figure 1E). In contrast to germ-line variation, selective deletion in *Famin*^ΔDC^ mice did not increase immunopathology (Figure S1D). Interestingly, the excessive IAV-specific T lymphocyte response in *Famin*^p.284R^ and *Famin*^ΔDC^ lungs was associated with somewhat higher expression of IAV M protein compared to their respective controls (Figures 1F and S1E). We concluded that reduced or absent FAMIN activity in DCs resulted in exaggerated hyperinflammatory IAV-specific CD8^+^ T cell responses that did not augment control of the viral infection.

### FAMIN in DCs restrains priming of class I and class II-restricted antigens

**Figure 2:**
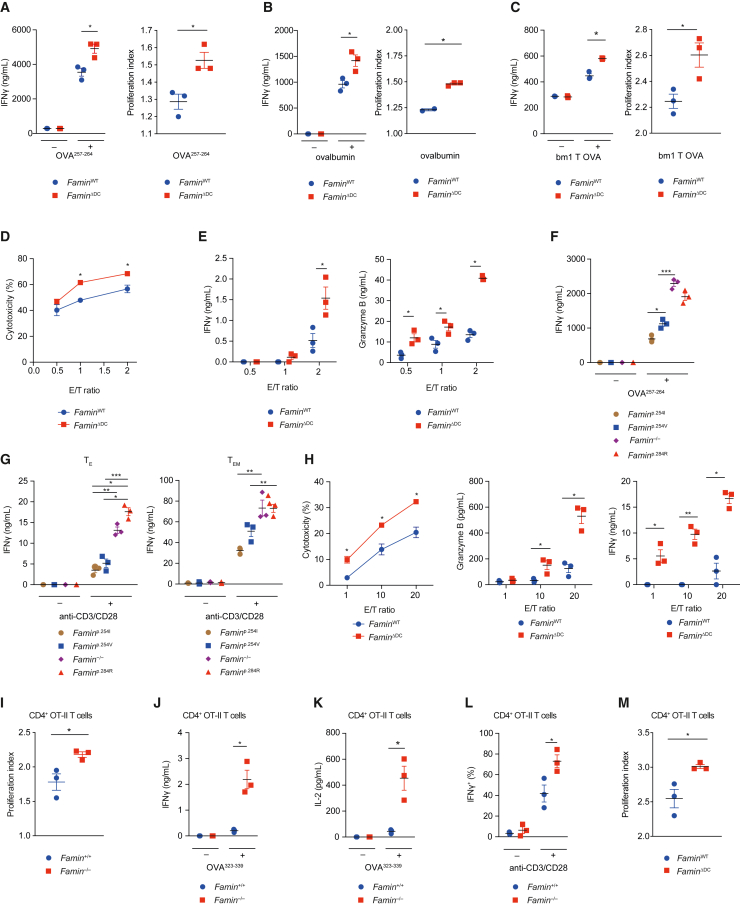
DC FAMIN restrains CD4^+^ and CD8^+^ T cell responses (A–C) IFNγ release and OT-I T cell proliferation indices after 72 h of co-culture with splenic *Famin*^WT^ or *Famin*^ΔDC^ CD11c^+^ DCs pulsed with OVA^257-264^ peptide (A), ovalbumin (B), or UV-irradiated bm1 T OVA mouse embryonic fibroblasts (C) (n = 3). (D and E) Specific cytotoxicity against OVA^257-264^-pulsed wild-type splenocytes (D), and IFNγ and granzyme B release (E) of OT-I T cells that had been primed with *Famin*^WT^ or *Famin*^ΔDC^ splenic DCs pulsed with OVA^257-264^ (n = 3). (F) IFNγ secretion from re-stimulated (OVA^257-264^ for 5 h) OT-I T cells after 72 h of priming with *Famin*^p.254I^, *Famin*^p.254V^, *Famin*^p.284R^, or *Famin*^−/−^ BM-derived cDC1 pulsed with OVA^257-264^ (n = 3). (G) IFNγ secretion after 5 h anti-CD3/CD28 re-stimulation of OT-I T cells that had been differentiated into T_E_ and T_EM_ cells, following 72 h of priming by *Famin*^p.254I^, *Famin*^p.254V^, *Famin*^p.284R^, and *Famin*^−/−^ BM-derived cDC1 pulsed with OVA^257-264^ (n = 3). (H) OVA^257-264^-specific cytotoxicity, granzyme B, and IFNγ secretion of splenocytes of *Famin*^ΔDC^ and *Famin*^WT^ mice that had been adoptively transferred with naive OT-I T cells and immunized with ovalbumin 72 h earlier (n = 3). (I) Proliferation indices of OT-II T cells 96 h after priming with OVA^323-339^-pulsed splenic *Famin*^+/+^ and *Famin*^−/−^ DCs (n = 3). (J and K) IFNγ (J) and IL-2 (K) in supernatants of OT-II cells 96 h after priming with OVA^323-339^-pulsed splenic *Famin*^+/+^ and *Famin*^−/−^ DCs (n = 3). (L) Percentage IFNγ^+^ OT-II T cells after restimulation with anti-CD3/CD28, following priming with OVA^323-339^-pulsed BM-derived cDC2 7 days earlier (n = 3). (M) Proliferation indices of OT-II T cells adoptively transferred into *Famin*^ΔDC^ and *Famin*^WT^ mice 72 h after immunization with ovalbumin (n = 3). Data represented as mean ± SEM. ^∗^p < 0.05, ^∗∗^p < 0.01, and ^∗∗∗^p < 0.001 (one-way ANOVA or unpaired two-tailed Student’s t test where appropriate). See also Figure S2.

Intrigued by the selective increase in T cell responses emanating from FAMIN deficiency in DCs, we focused our study on whether and how FAMIN controls T cell priming and turned to ovalbumin (OVA) as a model antigen. Baseline percentages of splenic and lymph node CD4^+^ and CD8^+^ T lymphocytes, and splenic cDC1s and cDC2s, were indistinguishable between *Famin*^p.254I^, *Famin*^p.254V^, and *Famin*^p.284R^ mice (Table S1). Splenic CD11c^+^ DCs from *Famin*^ΔDC^ mice primed naive OVA^257-264^-specific OT-I T lymphocytes for increased expansion and IFNγ secretion compared to those primed by *Famin*^WT^ DCs, irrespective of whether they were pulsed with OVA^257-264^ peptide, OVA protein, or necrotic fibroblasts expressing a non-secreted OVA (bm1T-OVA; Sancho et al., 2009) requiring cross-presentation (Figures 2A–2C). Antigen-specific cytotoxicity, IFNγ, and granzyme B release were higher when naive OT-I T cells had been primed by *Famin*^ΔDC^ than by *Famin*^WT^ splenic DCs (Figures 2D and 2E). CD8α^+^ conventional DCs type 1 (cDC1) preferentially prime naive CD8^+^ T cells, and CD11b^+^ cDC2 preferentially CD4^+^ T cells (Durai and Murphy, 2016). Exaggerated cytotoxic T lymphocyte (CTL) responses were similarly observed when primed by bone marrow (BM)-derived *Famin*^−/−^ compared to *Famin*^+/+^ cDC1 (Figure S2A), hence extending to DCs immunologically distinct from splenic DCs (Naik et al., 2005). BM-derived cDC1 from *Famin*^p.284R^ and *Famin*^−/−^ mice also primed OT-I T cells for higher proliferation and IFNγ secretion compared to those from *Famin*^p.254I^ and *Famin*^p.254V^ mice (Figure S2B). Restimulation of cDC1-OT-I T cell co-cultures after 72 h (Figure 2F), or after further differentiation over 6 days into antigen-specific T effector (T_E_) and T effector memory (T_EM_) cells via IL-2 and IL-15 (Figure 2G), respectively, resulted in highest IFNγ secretion when priming was provided by *Famin*^p.284R^ and *Famin*^−/−^ cDC1, intermediate by *Famin*^p.254V^, and lowest by *Famin*^p.254I^ cDC1. Hence, CD8^+^ T cell responses increased with decreasing FAMIN activity in DCs, and the enhanced priming effect persisted when further differentiated into T_E_ and T_EM_ cells.

To investigate this *in vivo*, OT-I T cells were adoptively transferred into *Famin*^ΔDC^ and *Famin*^WT^ mice followed by intraperitoneal immunization with ovalbumin, and CTL activity was assessed 4 days later. OVA^257-264^-specific cytotoxicity, IFNγ, and granzyme B release of splenic T cells were strikingly higher in *Famin*^ΔDC^ compared to *Famin*^WT^ mice (Figure 2H). Increased CTL activity was intrinsic, as OT-I proliferation *in vivo* was similar between genotypes upon adjuvant-free priming (Figure S2C). Hence, lack of FAMIN activity in DCs enhanced their ability to prime CD8^+^ T cell responses to a model antigen *in vivo*.

The ability of FAMIN-impaired DCs to prime exaggerated antigen-specific T cell responses extended to MHC II-restricted CD4^+^ T cells. *Famin*^−/−^ splenic CD11c^+^ DCs, pulsed with OVA^323-339^, primed for increased proliferation of syngeneic naive OVA^323-339^-specific OT-II T cells when compared to *Famin*^+/+^ DCs (Figure 2I). The levels of IFNγ and IL-2 were 4- and 8-fold higher, respectively, in supernatants from co-cultures with *Famin*^−/−^ compared to *Famin*^+/+^ DCs (Figures 2J and 2K). The proportion of intracellular IFNγ^+^ OT-II T cells upon restimulation increased from 40% ± 8% to 75% ± 6% when they had been primed by OVA^323-339^-pulsed *Famin*^−/−^ compared to *Famin*^+/+^ BM-derived cDC2 (Figure 2L). The proportion of IL-4^+^, IL-17^+^, and Foxp3^+^ OT-II T cells remained below 1% (Figure S2D). Naive CD4^+^ OT-II T cells adoptively transferred into *Famin*^ΔDC^ mice exhibited increased proliferation upon intraperitoneal OVA immunization compared to those transferred into *Famin*^WT^ mice (Figure 2M). Altogether, impaired FAMIN in DCs increased antigen-specific T cell priming via both class I and II *in vitro* and *in vivo*.

### FAMIN controls DC metabolism and tunes antigen uptake and presentation without a transcriptional signature

**Figure 3:**
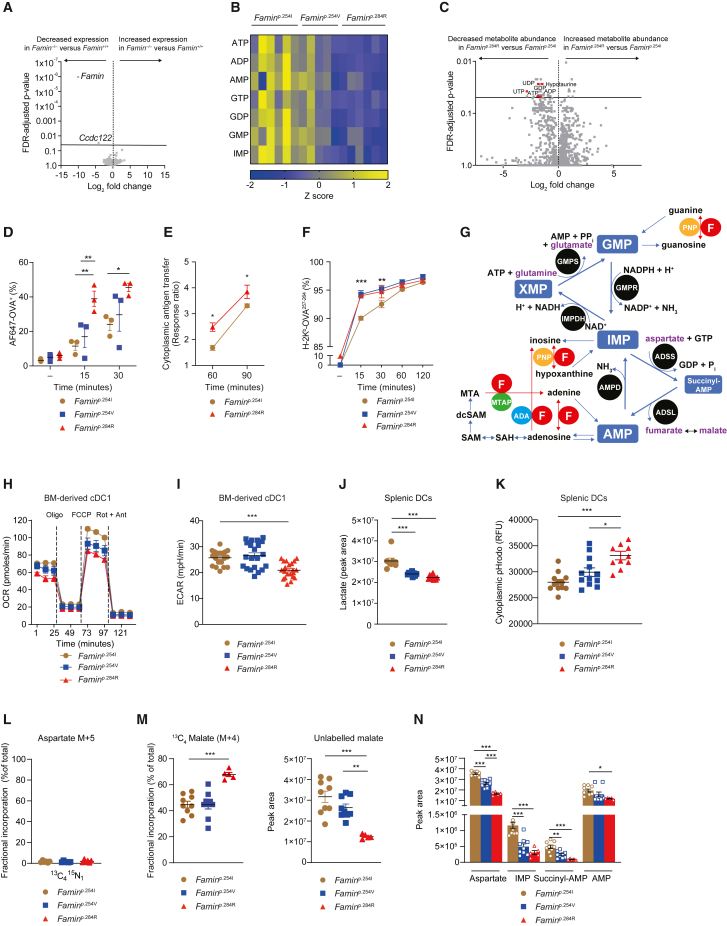
FAMIN controls DC metabolism and tunes antigen uptake and presentation without a transcriptional signature (A) Differentially expressed genes between *Famin*^−/−^ and *Famin*^+/+^ BM-derived cDC1s (n = 4; GEO: GSE126473). (B) Heatmap of purine nucleotide levels in *Famin*^p.254I^, *Famin*^p.254V^, and *Famin*^p.284R^ cDC1s (n = 6/5/6); for details, see Table S3. (C) Differential metabolite abundance between *Famin*^p.254I^ and *Famin*^p.284R^ BM-derived cDC1s; identifiable differential LC-MS features (positive and negative ionization modes) highlighted in red and annotated (n = 5). (D) Percentage AF647-OVA^+^*Famin*^p.254I^, *Famin*^p.254V^, or *Famin*^p.284R^ splenic CD11c^+^ DCs following uptake of AF647-OVA for indicated times (n = 3). (E) Endosome-to-cytosol transfer in β-lactamase-loaded *Famin*^p.254I^ and *Famin*^p.284R^ cDC1s (n = 6/6, from 3 mice per genotype). (F) Percentage splenic CD11c^+^ DCs staining positive for OVA^257-264^ bound to H-2K^b^, after incubation with OVA^257-264^ for indicated times (n = 3). (G) Schematic depiction of the IMP–S-AMP–AMP cycle and IMP–XMP–GMP cycle, with relationship to FAMIN products and substrates. ADSS, adenylosuccinate synthase; ADSL, adenylosuccinate lyase; AMPD, AMP deaminase; IMPDH, IMP dehydrogenase; GMPS, GMP synthase; GMPR, GMP reductase; HPRT, hypoxanthine guanine phosphoribosyltransferase; APRT, adenine phosphoribosyltransferase, ADA, adenosine deaminase; PNP, purine nucleoside phosphorylase; MTA, methylthioadenosine; MTAP, MTA phosphorylase; SAM, *S*-adenosylmethionine; dcSAM, decarboxylated SAM. (H) Oxygen consumption rate (OCR) of *Famin*^p.254I^, *Famin*^p.254V^, and *Famin*^p.284R^ BM-derived cDC1s. Basal OCR followed by oligomycin A (Oligo), FCCP, and rotenone plus antimycin A (Rot + Ant) (n = 16–18, from 3 mice per genotype). (I) Basal extracellular acidification rate (ECAR) of *Famin*^p.254I^, *Famin*^p.254V^, and *Famin*^p.284R^ BM-derived cDC1s (n = 16–18, from 3 mice per genotype). (J) Secreted lactate levels in supernatants of *Famin*^p.254I^, *Famin*^p.254V^, and *Famin*^p.284R^ splenic DCs after 3 h incubation in OptiMEM medium (n = 8–9, from 3 mice per genotype). (K) Cytoplasmic pH (pH_c_) of *Famin*^p.254I^, *Famin*^p.254V^, and *Famin*^p.284R^ splenic DCs measured using pHrodo indicator probe (n = 12, from 3 mice per genotype). (L) Fractional labeling of cellular aspartate as [^13^C_4_^15^N_1_] isotopomer in *Famin*^p.254I^, *Famin*^p.254V^, and *Famin*^p.284R^ BM-derived cDC1 following a 3 h pulse with [^13^C_4_ ^15^N_1_] aspartate (n = 6, from 3 mice per genotype). (M) Fractional labeling of [^13^C_4_] malate and levels of unlabeled (M+0) malate following a 3 h pulse of *Famin*^p.254I^, *Famin*^p.254V^, and *Famin*^p.284R^ cDC1s with [^13^C_4_] malate (n = 9/9/5, from 3/3/2 mice per genotype). (N) Total levels of aspartate, IMP, succinyl-AMP, and AMP following a 3 h pulse of *Famin*^p.254I^, *Famin*^p.254V^, and *Famin*^p.284R^ cDC1s with [^13^C_4_] malate (n = 9/9/5, from 3/3/2 mice per genotype). Data represented as mean ± SEM. ^∗^p < 0.05, ^∗∗^p < 0.01, and ^∗∗∗^p < 0.001 (one-way ANOVA or unpaired two-tailed Student’s t test where appropriate). See also Figure S3.

Despite the profound differences in their priming activity, *Famin* itself and genomically adjacent *Ccdc122* were the sole differentially expressed genes (DEGs) in *Famin*^−/−^ compared to *Famin*^+/+^ BM-derived cDC1 analyzed by RNA sequencing (RNA-seq; Figure 3A). A comparison of cDC1 from *Famin*^p.254I^ and *Famin*^p.254V^ mice did not reveal a single DEG (Figure S3A), and only 56 up- and 32 downregulated transcripts between *Famin*^p.284R^ and *Famin*^p.254I^ cDC1 (Figure S3B; Table S2). Among those DEGs were only four (*Fcgr1*, *Tlr7*, *Ikbkg*, and *Lcn2*) encoding immune mediators, and no enrichment for gene ontology processes linked to DC activation was observed (data not shown). *Famin* genotype did not affect protein expression of ADA, PNP, and MTAP (Figure S3C), which share catalytic activities with FAMIN. Liquid chromatography-mass spectrometry (LC-MS) demonstrated a marked reduction in purine nucleotides from *Famin*^p.254I^ via *Famin*^p.254V^ to *Famin*^p.284R^ cDC1 (Figures 3B and 3C; Table S3), raising the possibility of a bona fide biochemical mechanism controlling T cell priming.

Priming of naive T cells entails T cell receptor (TCR) binding to a peptide-MHC complex on a professional antigen-presenting cell, which is then fine-tuned by costimulatory molecules and secreted mediators (Blum et al., 2013; Cantrell, 2015). A pulse with a fluorescent AF647-ovalbumin conjugate (AF647-OVA) as model antigen demonstrated higher uptake, particularly at earlier time points, in *Famin*^p.284R^ compared to *Famin*^p.254I^ splenic DCs, with intermediate levels in *Famin*^p.254V^ cells (Figure 3D). Higher AF647-OVA uptake in *Famin*^p.284R^ and *Famin*^p.254V^ compared to *Famin*^p.254I^ genotypes was observed in both cDC1 and cDC2 splenic DCs, as well as in BM-derived cDCs (Figures S3D and S3E). This suggested that in the absence of FAMIN activity, antigen uptake was accelerated and available for presentation via MHC class II for CD4^+^ T cells and cross-presentation via MHC class I for CD8^+^ T cells, the latter requiring endosome-to-cytosol transfer (Blander, 2018). This can be measured using endocytosed β-lactamase in DCs that are pre-loaded with a cytosolic probe that loses its FRET signal upon β-lactamase cleavage, when the latter gains access to the cytosol (Cebrian et al., 2011). Compared to *Famin*^p.254I^ cells, *Famin*^p.284R^ splenic DCs exhibited increased probe cleavage, especially at the earliest time point, demonstrating increased endosome-to-cytosol transfer (Figure 3E). The peptide repertoire presented on surface MHC I is continuously optimized by peptide exchange in the endoplasmic reticulum (ER) (Williams et al., 2002). Increased staining with monoclonal antibody 25-D1.16, which recognizes OVA^257-264^ bound to H-2K^b^ (Porgador et al., 1997), directly demonstrated increased peptide presentation on *Famin*^p.284R^ and *Famin*^p.254V^ compared to *Famin*^p.254I^ splenic DCs after a pulse with OVA^257-264^ (Figures 3F and S3F). The difference between *Famin* genotypes in peptide:MHC I complexes was again most pronounced early after the OVA^257-264^ pulse, indicative of FAMIN controlling the pace of the process. *Famin* genotype did not affect total surface MHC I and II expression (Table S4; Figure S3G). Hence, loss of FAMIN activity led to faster-paced endosomal antigen uptake, transfer to cytosol, and peptide exchange and presentation on MHC I.

FAMIN can promote flux through a cycle that interconverts IMP to succinyl-AMP (S-AMP), AMP, and back to IMP via sequential activities of adenylosuccinate synthase (ADSS), adenylosuccinate lyase (ADSL), and AMP deaminase (AMPD) (Figure 3G) (Cader et al., 2020). In skeletal muscle and macrophages, the IMP–S-AMP–AMP cycle promotes energy metabolism and is referred to as purine nucleotide cycle (PNC) (Cader et al., 2020; Lowenstein and Tornheim, 1971). Cellular levels of IMP, S-AMP, and AMP decreased from *Famin*^p.254I^ and *Famin*^p.254V^ to *Famin*^p.284R^ BM-derived cDC1 (Figures 3B and S3H). Tracing [^13^C_16_] palmitate, we observed decreased flux into Krebs cycle metabolites α-ketoglutarate, succinate, and malate in *Famin*^p.254V^ and *Famin*^p.284R^ compared to *Famin*^p.254I^ BM-derived DC1s (Figure S3I). We also detected decreased onward flux into aspartate (which enters the IMP–S-AMP–AMP cycle) in *Famin*^p.284R^ DCs. A similar pattern in labeled Krebs cycle metabolites was present after a pulse with [^13^C_6_] glucose, where onward flux (via pyruvate dehydrogenase) into [^13^C_2_] aspartate decreased, and onward flux (via pyruvate carboxylase or malic enzyme) into [^13^C_3_] aspartate increased in FAMIN-impaired DCs (Figure S3I). In contrast, flux from [^13^C_5_^15^N_2_] glutamine into Krebs cycle metabolites and aspartate increased in FAMIN-impaired cDC1s (Figure S3I). Overall, this was consistent with perturbed fatty acid oxidation (FAO) and lipid carbon channeling into the PNC, as well as compensatory changes in glutamine metabolism, mirroring key observations in FAMIN-deficient macrophages (Cader et al., 2020). Consequently, cDC1s’ oxygen consumption rate (OCR), reflecting oxidative phosphorylation (OXPHOS), decreased from *Famin*^p.254I^ via *Famin*^p.254V^ to *Famin*^p.284R^ genotypes (Figure 3H). The extracellular acidification rate (ECAR) was also lower in *Famin*^p.284R^ compared to *Famin*^p.254I^ BM-derived cDC1s (Figure 3I), and secretion of lactate, with which protons (H^+^) are co-exported, correspondingly declined from *Famin*^p.254I^ to *Famin*^p.254V^ and *Famin*^p.284R^ DCs (Figure 3J). Corresponding observations were made in splenic DCs (Figure S3J), in which cDC2 predominate over cDC1 (Table S1). The cytoplasmic pH (pH_c_) of cDC1 (data not shown) and splenic DCs became more acidic as FAMIN activity decreased (Figure 3K). This demonstrated that FAMIN promotes DCs’ energy metabolism and prevents cytoplasmic acidification. By consuming aspartate and releasing its carbons as fumarate, which can be hydrated to malate, the IMP–S-AMP–AMP cycle can affect electron (*e*^−^) transfer between cytoplasm and mitochondria, which ensues via the malate-aspartate shuttle (Borst, 2020; Cader et al., 2020). The aspartate pool supplying the IMP–S-AMP–AMP cycle was inaccessible by exogenously supplied [^13^C_4_^15^N_1_] aspartate (Figure 3L), similar to most cells in culture (Birsoy et al., 2015). Fractional incorporation of exogenously provided [^13^C_4_] malate was strikingly higher in *Famin*^p.284R^ compared to *Famin*^p.254I^ and *Famin*^p.254V^ BM-derived DCs, as levels of unlabeled malate were conversely lowest in *Famin*^p.284R^ and highest in *Famin*^p.254I^ cells (Figures 3M and S3K). These differences in cellular malate, encompassing cytoplasmic and mitochondrial pools, corroborated that energy metabolism is pervasively perturbed in FAMIN-impaired DCs. Exogenous malate resulted in marked differences in levels of aspartate, IMP, S-AMP, and AMP between *Famin* genotypes (Figure 3N). Altogether this pointed, in analogy to macrophages (Cader et al., 2020), to the IMP–S-AMP–AMP cycle as an immediate biochemical effector of FAMIN catalysis.

### Adenine-guanine nucleotide interconversion paces antigen uptake and T cell priming

**Figure 4:**
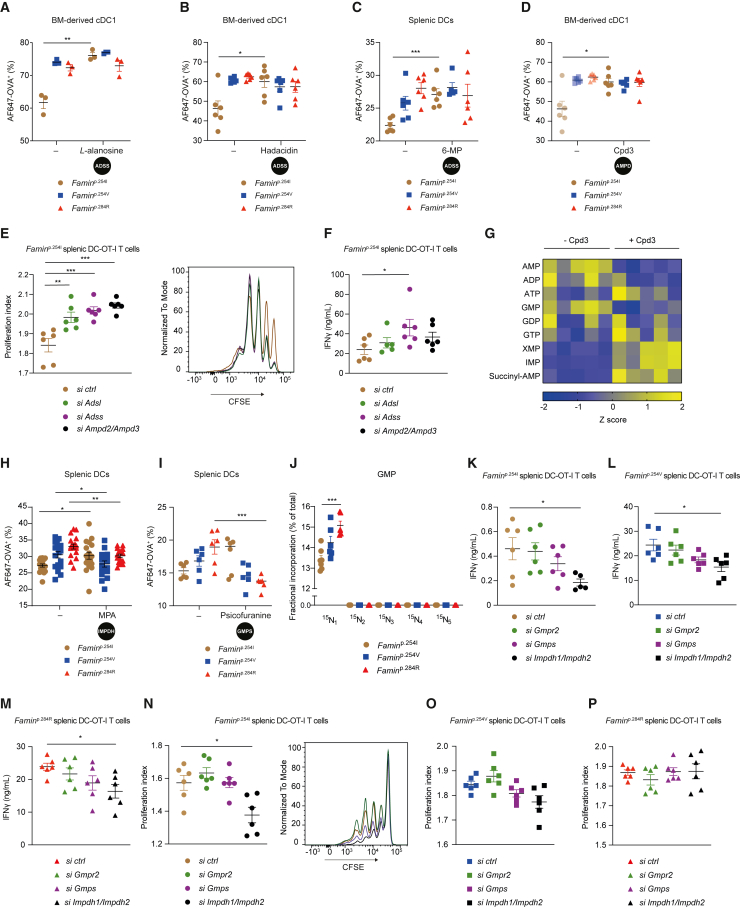
Adenine-guanine nucleotide interconversion paces antigen uptake and T cell priming (A–D) Percentage AF647-OVA^+^ cDC1s (A, B, and D) or splenic DCs (C) following incubation with AF647-OVA for 30 min in the presence of *L*-alanosine (A), hadacidin (B), 6-mercaptopurine (6-MP) (C), or Cpd3 (D) (n = 3–6, 3 mice per genotype; none of the treatments affected cell viability; please note control panels are shared between B and D). (E and F) Proliferation indices, CFSE overlays (E), and IFNγ released (F) from OT-I T cells co-cultured for 72 h with *Famin*^p.254I^ splenic DCs pulsed with ovalbumin 48 h after nucleofection with *ctrl* or *Adsl*, *Adss*, and *Ampd2/Ampd3* siRNAs (n = 6, 3 mice per siRNA). (G) Heatmap of purine nucleotide levels in *Famin*^p.254I^ cDC1s, treated with Cpd3 or vehicle for 18 h (n = 5); normalized peak integrals and fold changes are shown in Table S5. (H and I) Percentage AF647-OVA^+^ splenic DCs of indicated genotypes after incubation with mycophenolic acid (MPA) (H) or psicofuranine (I) (n = 18, from 3 independent experiments in H; n = 6, 3 mice per genotype in I). (J) Fractional incorporation into indicated GMP isotopomers following a 3 h pulse of *Famin*^p.254I^, *Famin*^p.254V^, and *Famin*^p.284R^ cDC1s with [^15^N_2_^13^C_5_] glutamine (n = 7/6/6, 3 mice per genotype). (K–M) IFNγ released from OT-I T cells co-cultured for 72 h with ovalbumin-pulsed *Famin*^p.254I^ (K), *Famin*^p.254V^ (L), or *Famin*^p.284R^ (M) splenic DCs 48 h after nucleofection with *Gmpr2*, *Gmps*, *Impdh1/Impdh2*, or *ctrl* siRNAs (n = 6, 3 mice per group). (N–P) Proliferation indices from OT-I T cells co-cultured for 72 h with *Famin*^p.254I^ (N), *Famin*^p.254V^ (O), and *Famin*^p.284R^ (P) splenic DCs pulsed with ovalbumin 48 h after nucleofection with *ctrl* or *Gmpr2*, *Gmps*, *Impdh1*, and *Impdh2* siRNA (n = 6, 3 mice per siRNA). Data represented as mean ± SEM. ^∗^p < 0.05, ^∗∗^p < 0.01, and ^∗∗∗^p < 0.001 (one-way ANOVA or unpaired two-tailed Student’s t test where appropriate). See also Figure S4.

We therefore asked whether IMP–S-AMP–AMP cycling restrains DC antigen uptake. Halting the IMP–S-AMP–AMP cycle with *L*-alanosine and hadacidin, IMP- and aspartate-analog inhibitors of ADSS (Guicherit et al., 1994), respectively, indeed increased AF647-OVA uptake in *Famin*^p.254I^ BM-derived cDC1 to levels observed in *Famin*^p.284R^ cells (Figures 4A and 4B). In contrast, blocking ADSS in *Famin*^p.284R^ cDC1 did not further increase AF647-OVA uptake (Figures 4A and 4B). Increased antigen uptake by *L*-alanosine, conditional on *Famin* genotype, was similarly observed in BM-derived cDC2 (Figure S4A). 6-thio-IMP, a metabolite of clinically used immunomodulators 6-mercaptopurine (6-MP) and azathioprine (Hanauer et al., 2019; Tiede et al., 2003), has been reported to inhibit ADSS (Atkinson et al., 1964). 6-MP phenocopied *L*-alanosine and hadacidin on *Famin*-dependent AF647-OVA uptake in splenic DCs (Figure 4C), affecting cDC1 and cDC2 subsets equally (Figure S4B). Transfection of splenic DCs with *Adss* small interfering RNA (siRNA) increased AF647-OVA uptake in *Famin*^p.254I^ cells to levels observed in control-transfected *Famin*^p.284R^ cells, while not further increasing uptake in the latter (Figure S4C). Inhibition of AMPD with Cpd3 (Admyre et al., 2014) recapitulated effects of ADSS inhibition (Figure 4D), corroborating that FAMIN-enabled IMP–S-AMP–AMP cycling restrains antigen uptake. We next assessed whether increased antigen uptake upon blocking the IMP–S-AMP–AMP cycle translates into enhanced T cell priming. Compared to control-silenced OVA-pulsed splenic *Famin*^p.254I^ DCs, those silenced for *Adss*, *Adsl*, or *Ampd2*/*Ampd3* primed naive OT-I T cells for increased proliferation, with IFNγ elevated upon *Adss* knockdown (Figures 4E, 4F, S4D, and S4E). Consistent with fatty acid carbon entering the IMP–S-AMP–AMP cycle (Cader et al., 2020; Figure S3I), silencing the rate-limiting enzyme of FAO, *Cpt1a*, in *Famin*^+/+^ DCs elevated their OT-I priming potency to levels observed in *Famin*^−/−^ cells, while not augmenting it further in the latter (Figure S4F). Hence, knockdown of IMP–S-AMP–AMP cycle enzymes in FAMIN-sufficient DCs, or blocking the upstream supply of carbon entering the cycle, phenocopied enhanced priming due to compromised FAMIN.

Membrane trafficking, including endocytosis and processes leading to antigen presentation, is controlled by proteins regulated by GTP/GDP binding (Kirschner and Mitchison, 1986; Stenmark, 2009). Guanine nucleotides are synthesized from IMP via xanthosine monophosphate (XMP), catalyzed by IMP dehydrogenase (IMPDH) and GMP synthase (GMPS; Figure 3G). GMP reductase (GMPR) converts GMP back to IMP (Figure 3G) (Hedstrom, 2012), which, together with the IMP–S-AMP–AMP cycle, interconverts adenine and guanine nucleotides. Cellular purine nucleotide levels all declined with compromised FAMIN activity, except XMP, which trended higher (Figure S4G). XMP also increased, and GMP and AMP decreased, upon AMPD inhibition in *Famin*^p.254I^ cDC1s (Figure 4G; Table S5). This suggested increased flux through IMPDH consequent to impaired FAMIN or IMP–S-AMP–AMP blockade. The IMPDH inhibitor mycophenolate (MPA), an immunosuppressant effective in arthritis and transplantation (Broen and van Laar, 2020), reduced AF647-OVA uptake in *Famin*^p.284R^ and *Famin*^p.254V^ splenic DCs (Figure 4H), as did the GMPS inhibitor psicofuranine (Udaka and Moyed, 1963) (Figure 4I), suggesting increased flux through GMPS, too. Increased flux through GMPS was evident by [^15^N_1_] GMP labeling increasing from *Famin*^p.254I^ to *Famin*^p.284R^ DCs after a pulse with [^13^C_5_^15^N_2_] glutamine (Figure 4J), from which the amide nitrogen is transferred to form GMP (Figures 3G and S4H) (Tesmer et al., 1996). No GMP isotopomers with [^15^N_2_] or higher were observed, which would have indicated *de novo* purine nucleotide synthesis (Figure S4H). We therefore tested whether increased flux through IMPDH and GMPS mediated enhanced priming. OVA-pulsed splenic DCs with *Impdh1/Impdh2* knockdown (Figures S4D and S4E) primed naive OT-I T cells for lower IFNγ secretion compared to control-silenced DCs (Figures 4K–4M), and lower proliferation, which declined from the different levels primed by *Famin*^p.254I^ and *Famin*^p.254V^ DCs (Figures 4N–4P). OT-I T cell IFNγ secretion and proliferation also trended lower when primed by *Gmps*-silenced splenic DCs (Figures 4K–4P). This demonstrated that increased flux via IMPDH and GMPS in FAMIN-impaired DCs was responsible for their increased antigen uptake and T cell priming.

### IMPDH-dependent NADH/NAD^+^ redox state controls the pace of antigen uptake and MHC I recycling

**Figure 5:**
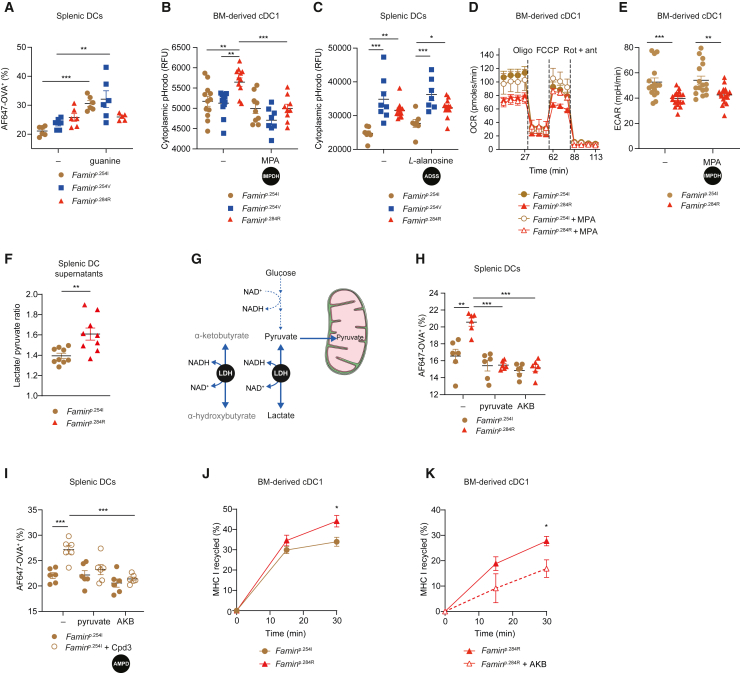
IMPDH-dependent NADH/NAD^+^ redox state controls the pace of antigen uptake and MHC I recycling (A) Percentage AF647-OVA^+^ splenic DCs of indicated genotypes following incubation with guanine (n = 6, 3 mice per genotype). (B) pH_c_ of *Famin*^p.254I^, *Famin*^p.254V^, and *Famin*^p.284R^ cDC1s in the presence of MPA (n = 8–12, 3 mice per genotype). (C) pH_c_ of *Famin*^p.254I^, *Famin*^p.254V^, and *Famin*^p.284R^ splenic DCs in the presence of *L*-alanosine (n = 9, 3 mice per genotype). (D) OCR of *Famin*^p.254I^ and *Famin*^p.284R^ BM-derived cDC1s in the presence of MPA or vehicle control. Basal OCR and OCR following addition of oligomycin A (Oligo), FCCP, and Rot + Ant (n = 7–14, 3 mice per genotype). (E) Basal ECAR of *Famin*^p.254I^ and *Famin*^p.284R^ BM-derived cDC1in the presence of MPA or vehicle control (n = 16–18, 3 mice per genotype). (F) Ratio of secreted lactate to pyruvate, reflective of free cytosolic NADH/NAD^+^, in supernatants of *Famin*^p.254I^ and *Famin*^p.284R^ splenic DCs matured overnight in RPMI-1640/10% FBS (n = 9, from 3 mice per genotype). (G) Schematic depicting the lactate dehydrogenase (LDH) reaction, in which pyruvate is converted to lactate with regeneration of NAD^+^; α-ketobutyrate acts as an alternative electron acceptor and is converted to α-hydroxybutyrate. (H) Percentage AF647-OVA^+^ splenic DCs of indicated genotypes following incubation with pyruvate or α-ketobutyrate (AKB) overnight and replenished for the time of the assay (n = 6, 3 mice per genotype). (I) Percentage AF647-OVA^+^*Famin*^p.254I^ splenic DCs following overnight incubation with pyruvate or AKB alone or in presence of Cpd3 (n = 6, 3 mice per genotype). (J) Percentage of MHC I recycled in *Famin*^p.254I^ and *Famin*^p.284R^ BM-derived cDC1s over time (n = 6, 3 mice per genotype). (K) Percentage of MHC I recycled in *Famin*^p.284R^ BM-derived cDC1s at indicated times following overnight incubation with AKB or control (n = 3–5, 3 mice per genotype). Data represented as mean ± SEM. ^∗^p < 0.05, ^∗∗^p < 0.01, and ^∗∗∗^p < 0.001 (one-way ANOVA or unpaired two-tailed Student’s t test where appropriate). See also Figure S5.

Bypassing IMPDH and GMPS with exogenous guanine increased AF647-OVA uptake in *Famin*^p.254I^ and *Famin*^p.254V^ splenic DCs, phenocopying enhanced antigen uptake of *Famin*^p.284R^ DCs (Figure 5A). In the latter, guanine did not further augment uptake (Figure 5A). *Prima vista*, this suggested that guanine nucleotide pools may control antigen uptake. Increased antigen uptake, however, was at odds with decreased GTP and GDP levels in FAMIN-impaired DCs (Figure 3B). This implied a byproduct of the IMP–XMP–GMP cycle, rather than guanine nucleotide pool size, may be responsible for increased membrane trafficking. IMPDH reduces NAD^+^ to NADH + H^+^ (Figure 3G). Inhibition of IMPDH rescued cytoplasmic acidification in *Famin*^p.284R^ DCs (Figure 5B), adding further evidence for enhanced flux through IMPDH. An altered pH_c_ can affect vesicular trafficking (Heuser, 1989; Korolchuk et al., 2011; Walton et al., 2018). ADSS inhibition in *Famin*^p.254I^ DCs enhanced AF647-OVA uptake (Figure 4A) without causing cytoplasmic acidification (Figure 5C), arguing against pH_c_ changes accounting for altered membrane trafficking. IMPDH inhibition did not rescue OCR or ECAR deficits in *Famin*^p.284R^ DCs (Figures 5D and 5E), confirming that compromised OXPHOS and glycolysis are not directly responsible for exaggerated antigen uptake. As total cellular NAD(H) integrates protein-bound and free forms across cytoplasmic and mitochondrial pools with their distinct redox states, we measured the secreted lactate/pyruvate ratio to deduce the cytosolic free NADH/NAD^+^ ratio (Goodman et al., 2020; Krebs, 1967; Williamson et al., 1967). *Famin*^p.284R^ splenic DCs exhibited a markedly higher lactate/pyruvate ratio than *Famin*^p.254I^ cells (Figure 5F), implying increased cytosolic NADH/NAD^+^. In contrast, the secreted β-hydroxybutyrate/acetoacetate ratio, reflecting mitochondrial free NADH/NAD^+^, remained unchanged (Figure S5A). To investigate whether re-oxidation of cytoplasmic NADH rescues exaggerated antigen uptake, we provided pyruvate as external *e*^−^ acceptor that regenerates NAD^+^ via lactate dehydrogenase (LDH) (Figure 5G). Pyruvate indeed rescued increased AF647-OVA uptake in *Famin*^p.284R^ splenic DCs (Figure 5H). Four-carbon α-ketobutyrate (AKB) is an alternative substrate for regenerating NAD^+^ from NADH via LDH (Figure 5G) (Sullivan et al., 2015). AKB is primarily used as *e*^−^ acceptor and not as carbon substrate in other metabolic pathways. AKB precisely phenocopied the rescue of antigen uptake achieved by pyruvate, decreasing AF647-OVA uptake to equally low levels in *Famin*^p.254I^ and *Famin*^p.284R^ splenic DCs (Figure 5H). AKB reduced the secreted lactate/pyruvate ratio as expected (Figure S5B), while the pH_c_ difference was retained between *Famin*^p.284R^ and *Famin*^p.254I^ splenic DCs (Figure S5C), consistent with cytoplasmic acidification not accounting for increased antigen uptake. Augmentation of baseline OCR was markedly different between pyruvate and AKB (Figures S5D and S5E). This was consistent with only pyruvate entering mitochondrial oxidation (Figure 5G), which we confirmed by tracing [^13^C_3_] pyruvate and [^13^C_4_] AKB into Krebs cycle metabolites in *Famin*^p.254I^ and *Famin*^p.284R^ cDC1s (Figures S5F–S5I). Importantly, pyruvate and AKB also rescued increased AF647-OVA uptake in *Famin*^p.254I^ splenic DCs, in which the IMP–S-AMP–AMP cycle was halted by Cpd3 (Figure 5I). AF647-OVA uptake serves as proxy of one specialized endocytic pathway, but membrane trafficking is involved in all the different routes of antigen uptake, processing, and (cross-)presentation (Alloatti et al., 2016; Blander, 2018). Considering whether FAMIN-mediated redox control of membrane trafficking is a more general principle, we asked whether FAMIN affects MHC I recycling, required for loading of cross-presented peptides (Belabed et al., 2020; Joffre et al., 2012). MHC I recycling was higher in *Famin*^p.284R^ compared to *Famin*^p.254I^ cDC1 (Figure 5J). AKB markedly reduced MHC I recycling in *Famin*^p.284R^, and barely in *Famin*^p.254I^ cDC1 (Figures 5K and S5J), revealing that cytoplasmic NADH/NAD^+^ affects the pace of MHC I recycling, too. This provided strong evidence that enhanced antigen uptake and presentation in FAMIN-impaired DCs is caused by increased reduction of cytoplasmic NAD^+^ to NADH by IMPDH due to an imbalance in adenine-guanine nucleotide interconversion cycles.

### The FAMIN product inosine dampens T cell activation during priming

**Figure 6:**
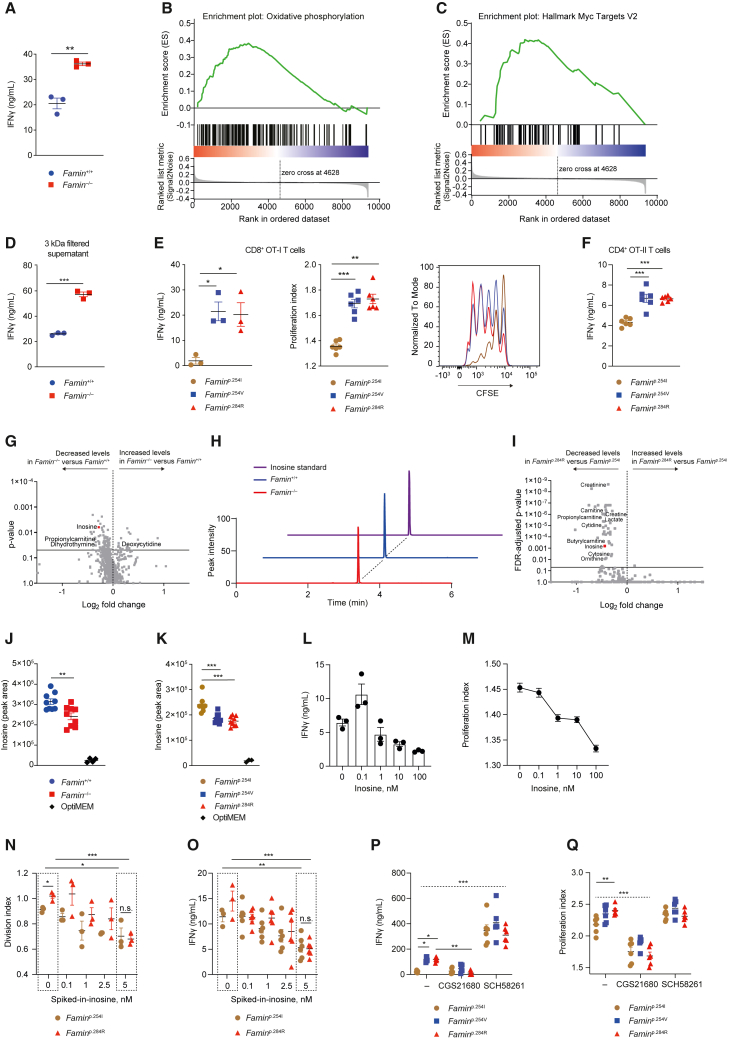
The FAMIN catalytic product inosine released from DCs dampens T cell activation during priming (A) IFNγ secretion from naive OT-I T cells activated by anti-CD3/CD28 in presence of 24 h supernatants of *Famin*^−/−^ or *Famin*^+/+^ CD11c^+^ splenic DCs (n = 3). (B and C) Gene set enrichment analysis (GSEA) of RNA-seq dataset of naive OT-I T cells activated by anti-CD3/CD28 for 24 h in the presence of *Famin*^−/−^ or *Famin*^+/+^ splenic DC supernatant; data depict enrichment of the Hallmark gene sets “oxidative phosphorylation” (B) and “Myc Targets V2” (C). (D) IFNγ secretion from OT-I T cells activated by anti-CD3/CD28 for 72 h in the presence of <3 kDa cut-off filtrates of supernatants from *Famin*^+/+^ or *Famin*^−/−^ splenic DCs cultured for 3 h in RPMI-1640/10% FBS (n = 3). (E) IFNγ secretion, proliferation indices, and CFSE overlays from OT-I T cells activated with anti-CD3/CD28 for 72 h in the presence of supernatants from *Famin*^p.254I^, *Famin*^p.254V^, and *Famin*^p.284R^ splenic DCs cultured for 3 h in OptiMEM (n = 3). (F) IFNγ from 72 h anti-CD3/CD28 stimulated naive OT-II T cells cultured in the presence of 3 h supernatant from *Famin*^p.254I^, *Famin*^p.254V^, and *Famin*^p.284R^ splenic DCs (n = 6, 3 mice per genotype). (G) Differential abundance of LC-MS features in supernatants from *Famin*^−/−^ versus *Famin*^+/+^ splenic DCs cultured for 3 h in OptiMEM (n = 8–9, 3 mice per genotype). Data depicted as volcano plot showing p value and log_2_ fold change for each detected LC-MS feature. (H) Representative extracted chromatograms, using normalized peak intensity, showing inosine detection in supernatants from *Famin*^−/−^ and *Famin*^+/+^ splenic DCs and corresponding standard. (I) Differential LC-MS features in supernatants from *Famin*^p.254I^ and *Famin*^p.284R^ splenic DCs cultured for 3 h in OptiMEM (n = 8–9, 3 mice per genotype). Data depicted as volcano plot showing p value and log_2_ fold change for each detected LC-MS feature; FDR-adjusted p value is depicted. (J and K) Relative inosine levels released in supernatants from *Famin*^−/−^ or *Famin*^+/+^ (J) or *Famin*^p.254I^, *Famin*^p.254V^, and *Famin*^p.284R^ (K) splenic DCs, cultured for 3 h in OptiMEM (n = 8–9, 3 mice per genotype). (L and M) IFNγ secretion (L) and proliferation indices (M) of naive OT-I T cells stimulated with anti-CD3/CD28 in the presence of indicated concentrations of inosine for 72 h (n = 3). (N and O) Division index (N) and IFNγ secretion (O) from *Famin*^p.254I^ and *Famin*^p.284R^ splenic DCs pulsed with ovalbumin and co-cultured with OT-I T cells in presence of indicated spiked-in concentrations of inosine into RPMI-1640 (concentration from 0.1 to 5 nM, with 5 nM reflecting the lower end of differences in inosine release between *Famin*^p.254I^ and *Famin*^p.284R^ DC supernatants, such as in Figure S6H) (n = 3–6, from 3 mice per genotype). (P and Q) IFNγ secretion (P) and proliferation indices (Q) of OT-I T cells activated by anti-CD3/CD28 for 72 h in the presence of CGS21680 or SCH58261 and supernatants from *Famin*^p.254I^, *Famin*^p.254V^, and *Famin*^p.284R^ splenic DCs cultured for 3 h in OptiMEM (n = 6, 3 mice per genotype). Data represented as mean ± SEM. ^∗^p < 0.05, ^∗∗^p < 0.01, and ^∗∗∗^p < 0.001 (one-way ANOVA or unpaired two-tailed Student’s t test where appropriate). See also Figure S6.

Fixing splenic DCs with glutaraldehyde after pulsing with OVA^257-264^ retained the ability of *Famin*^p.284R^ compared to *Famin*^p.254I^ DCs to prime for increased OT-I T cell proliferation (Figure S6A), but the capacity to prime for enhanced IFNγ secretion by *Famin*^p.284R^ DCs, however, was lost (Figure S6B). This indicated that optimal priming requires mutual dynamic transmembrane signaling. It also raised the possibility that soluble factors released from DCs might be involved, too. *Famin*^−/−^ and *Famin*^+/+^ DCs, and *Famin*^p.254I^, *Famin*^p.254V^, and *Famin*^p.284R^ cDC1s, were indistinguishable in their expression of co-stimulatory and co-inhibitory molecules (Table S4; Figure S6C). Cell-free supernatants of *Famin*^−/−^ DCs primed naive anti-CD3/CD28-activated OT-I T cells to secrete 2-fold more IFNγ compared to supernatants of *Famin*^+/+^ DCs (Figure 6A). Transcriptomes of OT-I T cells activated in the presence of *Famin*^−/−^ compared to *Famin*^+/+^ DC supernatants were enriched for hallmark gene sets (Leone et al., 2019) indicative of elevated effector function (Figures 6B, 6C, S6D, and S6E; Table S6). IL-12p70 and IFNα secretion by *Famin*^−/−^ and *Famin*^+/+^ DCs in co-culture with naive OT-I T cells was indistinguishable (Figure S6F). Freeze-thaw cycles did not affect *Famin*^−/−^ DC supernatants’ enhanced stimulatory capacity (data not shown), which was retained after passing through a 3 kDa filter (Figure 6D), pointing to a small molecule. To enable its identification, we switched to serum-free OptiMEM media to reduce complexity. OptiMEM supernatants of *Famin*^−/−^ and *Famin*^+/+^ DCs retained differences in IFNγ induction in anti-CD3/CD28-activated CD8^+^ T cells (Figure S6G). They were particularly stark between those elicited by *Famin*^p.254I^ compared to *Famin*^p.254V^ DC supernatants (Figure 6E). CD4^+^ OT-II T cells were also primed for heightened IFNγ secretion by *Famin*^p.254V^ and *Famin*^p.284R^ compared to *Famin*^p.254I^ DC supernatants (Figure 6F). This suggested that DCs secrete a small molecule in a FAMIN-dependent manner that inhibits priming of naive CD4^+^ and CD8^+^ T cells.

Unbiased high-resolution LC-MS of supernatants of *Famin*^+/+^ and *Famin*^−/−^ splenic DCs resolved ∼1,100 features, revealing inosine as the top-ranking identifiable LC-MS feature of differential abundance (Figures 6G and 6H). A second unbiased LC-MS screen comparing *Famin*^p.254I^ with *Famin*^p.284R^ splenic DC supernatants identified two metabolites, inosine and propionyl-carnitine, overlapping with the first screen (Figure 6I). As a catalytic product of FAMIN, inosine, whose levels were highest with fully active FAMIN-254I (Figures 6J and 6K), was a plausible candidate. Pure inosine dose-dependently reduced OT-I T cell IFNγ secretion and proliferation after anti-CD3/CD28 stimulation (Figures 6L and 6M). Spiking co-cultures of OVA-presenting splenic DCs and naive OT-I T cells with just 5 nM inosine, the lower end of differentials in inosine levels between *Famin*^p.254I^ and *Famin*^p.284R^ DC supernatants (Figure S6H), was sufficient to decrease T cell proliferation and IFNγ secretion (Figures 6N and 6O). This suggested inosine as an inhibitory signal during T cell priming. Among the four adenosine receptors, direct binding of, and activation by, inosine has been shown for A_2A_R (Welihinda et al., 2018), the only adenosine receptor expressed on T cells (Cekic et al., 2013). In OT-I T cells activated by anti-CD3/CD28 in the presence of DC supernatants, the A_2A_R agonist CGS21680 (Hutchison et al., 1989) inhibited proliferation and IFNγ secretion and abrogated *Famin* genotype-related differences (Figures 6P and 6Q). A_2A_R antagonism with SCH58261 (Zocchi et al., 1996) had the converse effect (Figures 6P and 6Q). This demonstrated that FAMIN-dependent release of inosine from DCs inhibited activation of naive T cells.

### FAMIN-dependent conversion of extracellular hypoxanthine into inosine

**Figure 7:**
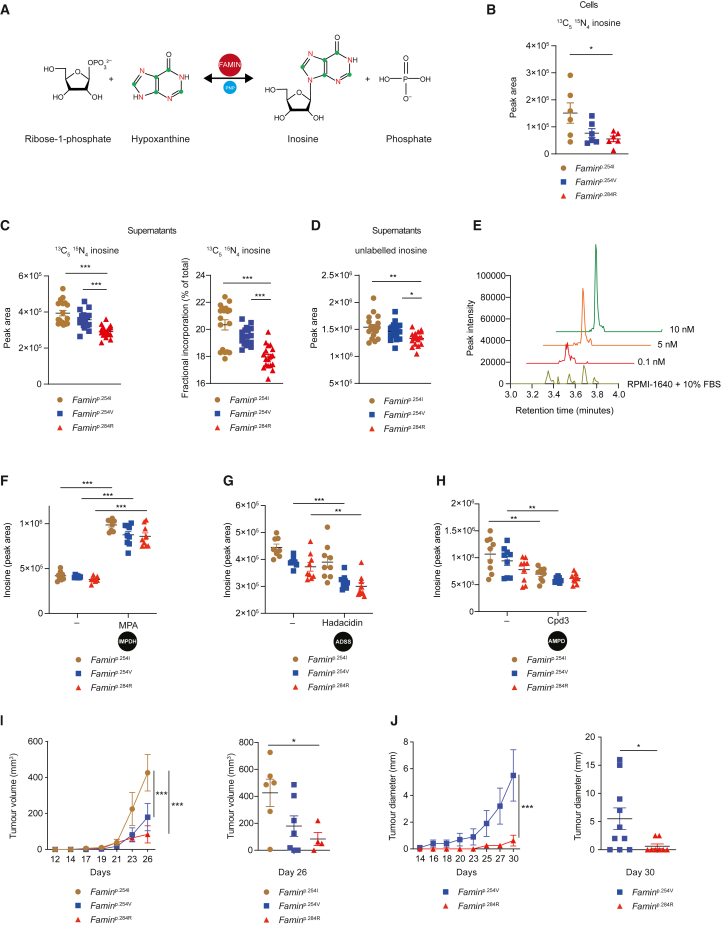
FAMIN-dependent conversion of extracellular hypoxanthine into inosine (A) FAMIN-catalyzed enzymatic conversion of [^13^C_5_^15^N_4_] hypoxanthine to [^13^C_5_^15^N_4_] inosine. (B) Cellular [^13^C_5_^15^N_4_] inosine in *Famin*^p.254I^, *Famin*^p.254V^, and *Famin*^p.284R^ splenic DCs pre-equilibrated in OptiMEM for 3 h before a 3 h pulse with [^13^C_5_^15^N_4_] hypoxanthine in OptiMEM (n = 6, 3 mice per genotype). (C) [^13^C_5_^15^N_4_] inosine, and its fractional incorporation, in supernatants of *Famin*^p.254I^, *Famin*^p.254V^, and *Famin*^p.284R^ splenic DCs pre-equilibrated in OptiMEM for 3 h before a 3 h pulse with [^13^C_5_^15^N_4_] hypoxanthine in OptiMEM (n = 18, 3 mice per genotype). (D) Unlabeled (M+0) inosine in supernatants of *Famin*^p.254I^, *Famin*^p.254V^, and *Famin*^p.284R^ splenic DCs following a 3 h pulse with [^13^C_5_^15^N_4_] hypoxanthine in OptiMEM (n = 18, 3 mice per genotype). (E) Representative extracted chromatograms, showing peak corresponding to inosine for indicated standards and for RPMI-1640/10% FBS medium, in which inosine was below the quantification limit. (F–H) Inosine levels released into supernatants from *Famin*^p.254I^, *Famin*^p.254V^, and *Famin*^p.284R^ splenic DCs, pre-treated with MPA (F), hadacidin (G), or Cpd3 (H) for 18 h and cultured for 3 h in OptiMEM with inhibitors replenished in medium (n = 9, 3 mice per genotype). (I) Tumor volume over time, and on day 26, after subcutaneous inoculation of *Famin*^p.254I^, *Famin*^p.254V^, and *Famin*^p.284R^ mice with 2.5 × 10^4^ LL2-OVA cells (n = 6/7/7, three mice were sacrificed due to fight wounds on day 23 during the blinded phase of the experiment; genotype was post hoc identified as *Famin*^p.284R^). (J) Tumor diameter over time and on day 30 post inoculation of *Famin*^p.254V^ and *Famin*^p.284R^ mice subcutaneously injected with 2 × 10^4^ LL2-OVA cells (n = 10/8). Data represented as mean ± SEM. ^∗^p < 0.05, ^∗∗^p < 0.01, and ^∗∗∗^p < 0.001 (one-way ANOVA or unpaired two-tailed Student’s t test where appropriate). See also Figure S7.

Nucleobases and nucleosides equilibrate across the plasma membrane via purine/pyrimidine transporters (Boswell-Casteel and Hays, 2017), prompting us to consider whether external nucleobases may supply the substrate for the synthesis of inosine in DCs (Figure 7A). A 3 h pulse of splenic DCs with 25 μM [^13^C_5_^15^N_4_] hypoxanthine labeled over half of extracellular and cellular hypoxanthine (Figures S7A–S7D). Labeled hypoxanthine was converted to cellular [^13^C_5_^15^N_4_] inosine, which was highest in *Famin*^p.254I^ and lowest in *Famin*^p.284R^ DCs (Figures 7B and S7E). This resulted in [^13^C_5_^15^N_4_] inosine released into supernatants, whose levels were, in relative terms, ∼35% and ∼22% higher in *Famin*^p.254I^ and *Famin*^p.254V^, respectively, than in *Famin*^p.284R^ DCs (Figure 7C). Even fractional [^13^C_5_^15^N_4_] inosine labeling increased from *Famin*^p.284R^ to *Famin*^p.254V^ and *Famin*^p.254I^ supernatants, averaging at one-fifth of total (Figure 7C), despite unlabeled inosine levels increased alongside, too (Figure 7D). Since OptiMEM is inosine-free and levels in RPMI-1640/10% FBS negligible (<100 pM, below the lowest detectable standard; Figure 7E), media change for labeling studies prompt an immediate equilibrative efflux; hence, these marked differences likely underestimate the contribution of FAMIN to inosine release during priming *in situ*. Plasma levels of inosine were similar across *Famin* germline mutants and DC-selective deletion (Figures S7F and S7G), consistent with a model of localized release. Perturbation in adenine-guanine nucleotide interconversion revealed increased inosine release upon IMPDH inhibition and a slight decrease upon ADSS and AMPD inhibition, with differential release across *Famin* genotypes remaining intact (Figures 7F–7H). Altogether these studies demonstrated that elevated inosine release by DCs with active FAMIN amplifies an inhibitory signal during T cell priming, generated by phosphoribosylation of largely extracellularly derived hypoxanthine.

### Compromised FAMIN catalysis enhances tumor immune surveillance

We finally turned to tumor immunosurveillance, a model not confounded by increased viral replication associated with IMPDH activity (To et al., 2016), to assess endogenous CTL function primed in the setting of polymorphic *Famin* variants. Increased DC antigen presentation enhances tumor-specific immunity by inducing Th1 and CTL responses (Xia et al., 2018). A_2A_R signaling prevents T cell anti-tumor immunity by inhibiting CTL activation and maintaining naive T cells quiescent (Cekic et al., 2013; Ohta et al., 2006). *Famin*^p.254I^ and *Famin*^p.254V^ mice developed markedly larger tumors compared to *Famin*^p.284R^ mice (Figures 7I and 7J) when subcutaneously injected with a syngeneic Lewis lung carcinoma cell line expressing ovalbumin (LL/2-OVA) (Kraman et al., 2010). Protection was associated with nominally higher OVA^257-264^-specific CD8^+^ T cell numbers in peripheral blood in *Famin*^p.284R^ compared to *Famin*^p.254I^ and *Famin*^p.254V^ mice (Figure S7H). These results were consistent with augmented priming that translated into increased CTL activity and tumor immunosurveillance when FAMIN activity is compromised.

## Discussion

Here we discovered that purine nucleotide and nucleoside turnover in DCs represses T cell immunity, with FAMIN acting as a purely biochemical immune checkpoint. FAMIN achieves this via two main routes. First, FAMIN restrains endocytosis, antigen processing, and presentation via cytoplasmic NADH/NAD^+^ through balancing adenine-guanine nucleotide interconversion. Second, it amplifies an inhibitory signal through the generation of locally released inosine. The relative contribution of altered antigen presentation versus altered inosine release to T cell priming is impossible to disentangle, since both are directly catalytically controlled by FAMIN. ADA, PNP, and MTAP do not compensate for FAMIN’s absence, although they share three of four of FAMIN’s catalytic activities. This suggests that FAMIN is at the center of a dedicated purine metabolon that biochemically restrains DCs’ priming activity.

Pacing membrane trafficking via adenine-guanine nucleotide interconversion cycles through an NADH/NAD^+^-sensitive mechanism, consequent to hyperactive IMPDH that reduces NAD^+^ to NADH, may represent a general principle. Vectorial physical membrane displacements, which can be energized by transmembrane *e*^−^ transport, occur upon plasma membrane internalization and, in the opposite direction, during membrane budding and vesicle formation (Morré and Morré, 2011). NADH can activate vectorial membrane transfer, elegantly demonstrated in cell-free systems for the transfer from the *trans* Golgi apparatus to the plasma membrane (Rodriguez et al., 1992). Aside from IMPDH, NAD^+^ is reduced to NADH by several cytoplasmic reactions, foremost by glyceraldehyde dehydrogenase of glycolysis, and re-oxidized by LDH and the malate-aspartate shuttle via mitochondria. NADH/NAD^+^ reductive stress due to perturbation in any of those reactions might therefore also impact membrane trafficking and antigen presentation. NADH/NAD^+^ reductive stress in the liver emerges as the causal mechanism for features of the metabolic syndrome associated with hypomorphic *GCKR*, such as hepatic insulin resistance and increased triglyceride release (Goodman et al., 2020). *GCKR* localizes at the probably most pleiotropic genome-wide association study (GWAS) locus and encodes liver-specific glucokinase regulatory protein, which helps prevent a futile metabolic cycle with glycolysis during gluconeogenesis (Goodman et al., 2020). Whether obesity, which increases risk for autoimmunity (Versini et al., 2014), causes NADH/NAD^+^ reductive stress in DCs is unknown.

Via equilibration of purine nucleobases and nucleosides across the plasma membrane, DCs may survey their vicinity. They respond to hypoxanthine by converting it to inosine, dampening T cell activation. Since phosphorolysis is reversible, DCs might bidirectionally respond to local hypoxanthine and inosine availability during immunological synapse formation. T cells, in particular naive CD4^+^ T cells, can release hypoxanthine (Fan et al., 2019), which may enable a dynamic interaction with FAMIN catalysis in DCs affecting the priming threshold. Systemic hypoxanthine and inosine levels closely track each other, consistent with their rapid interconversion via PNP (Sun et al., 2019). The intestinal microbiota may also affect inosine plasma levels (Mager et al., 2020). Inosine activation of the A_2A_R on T cells is itself complex: inosine either prevented Th1 differentiation and blunted anti-tumor immunity in anti-CTLA4-treated mice or, conversely, enhanced both, when co-supplied with IFNγ *in vitro* and a TLR9 agonist *in vivo* (Mager et al., 2020). The mechanism underlying this switch remained unclear. Inosine can also serve as an alternative carbon source for CD8^+^ T cells when glucose is unavailable (Wang et al., 2020). The complete (254I versus 254V) and almost-complete (254I versus 284R and *Famin*^*+/+*^ versus *Famin*^−/−^) absence of transcriptomic changes excludes that autocrine inosine-triggered receptor signaling in DCs dampens their priming capacity.

The biochemical mechanism reported herein sheds new light on predisposition for HLH/MAS. Hyperactivated CTLs and their IFNγ activate macrophages, triggering hemophagocytosis and a cytokine storm (Brisse et al., 2016b). Persistence of antigen-presenting DCs resulting in uncontrolled CTL priming occurs in primary, genetic HLH, consequent to impaired perforin-mediated antigen-selective removal of DCs (Lykens et al., 2011). However, most patients with secondary, acquired HLH have unimpaired cytotoxicity (Bryceson et al., 2012). Acquired HLH/MAS complicates diverse, mostly viral, infections, malignancies, autoimmune and autoinflammatory disorders (Brisse et al., 2016a), and treatment with chimeric antigen receptor (CAR) T cells (Neelapu et al., 2018). Five of six candidate genes at the major 3p21.31 risk locus for severe COVID-19 (Ellinghaus et al., 2020; Nakanishi et al., 2021; Pairo-Castineira et al., 2021) point toward DC-T cell interactions (Kaser, 2020), remarkable as that hyperinflammation shares features with HLH/MAS, including hemophagocytosis (Lucas et al., 2020; Prieto-Pérez et al., 2020). It remains unclear why excessive T cell activation by FAMIN-impaired DCs results in enhanced immunosurveillance of tumors but fails to control IAV infection.

A final point is that experiments in wild-type mice may grossly over-estimate anti-viral and anti-tumor T cell immunity that can be expected in the majority of humans, since mice naturally express hypomorphic FAMIN-254V. As we show here, the single amino acid change to −254I (for which ∼94% of humans are homozygous or heterozygous) results in 4-fold lower numbers of nucleoprotein-specific CTLs upon experimental IAV infection and a profound reduction in T cell effector function. This polymorphism (rs3764147) has been linked to a possible founder effect (Rivas et al., 2018), hinting that excessive priming may have afforded evolutionary benefits.

### Limitations of study

The IMP–S-AMP–AMP cycle operates at the center of energy metabolism, directly and instantaneously affecting glycolysis, electron transfer, FAO, Krebs cycle activity, glutamine oxidation, and the urea cycle (Cader et al., 2020; Lowenstein, 1972, 1990). The lack of technology with temporo-spatial resolution to resolve metabolites across cellular compartments is a particularly acute limitation, compounded by the unparalleled degree of interconnectedness, fast substrate cycles, and redundancies within central purine metabolism. This poses challenges, e.g., for directly measuring flux through IMPDH, and for determining inosine levels at the immunological synapse *in situ*.

## STAR★Methods

### Key resources table

**Table:** 

REAGENT or RESOURCE	SOURCE	IDENTIFIER
**Antibodies**		

B220-PE	Biolegend	RRID: AB_312992
CD11b-Pe/Cy7	Biolegend	RRID: AB_312799
CD11c-APC	Biolegend	RRID: AB_313778
CD11c-PE	Biolegend	RRID: AB_313777
CD16/CD32	Biolegend	RRID: AB_312801
CD172a (SIRPα)-PE/Dazzle 594	Biolegend	RRID: AB_2565279
CD24-AF647	Biolegend	RRID: AB_493485
CD252-Pe/Cy7	Biolegend	RRID: AB_2565744
CD274-BV785	Biolegend	RRID: AB_2629659
CD275-PE	Biolegend	RRID: AB_2248797
CD28 (37.51)	Thermo Fisher Scientific	RRID: AB_468921
CD3-AF647	Biolegend	RRID: AB_389323
CD3-PE/Cy5	Biolegend	RRID: AB_312674
CD3-PECy7	Biolegend	RRID: AB_312675
CD3ε (145-2C11)	Thermo Fisher Scientific	RRID: AB_468848
CD4-BV605	Biolegend	RRID: AB_2564591
CD4-FITC	Biolegend	RRID: AB_312691
CD40-PE/Cy7	Biolegend	RRID: AB_10933422
CD44-FITC	Biolegend	RRID: AB_493684
CD45-APC/Cy7	Biolegend	RRID: AB_312980
CD45-Pe/Cy7	Biolegend	RRID: AB_312978
CD45.1-APC	Biolegend	RRID: AB_313503
CD45R/B220-FITC	Biolegend	RRID: AB_312990
CD64-BV605	Biolegend	RRID: AB_2629778
CD8α-BV605	Biolegend	RRID: AB_2561352
CD8α-BV650	Biolegend	RRID: AB_11124344
CD80-AF647	Biolegend	RRID: AB_492824
CD86-FITC	Biolegend	RRID: AB_313149
Foxp3-PerCP/Cy5.5	Thermo Fisher Scientific	RRID: AB_914349
H-2K^b^-PerCP/Cy5.5	Biolegend	RRID: AB_1967107
H-2Kb-SIINFEKL (25-D1.16)-APC	Biolegend	RRID: AB_11219402
H-2K^b^/H-2D^b^-FITC	Biolegend	RRID: AB_313597
I-A/I-E-APC/Cy7	Biolegend	RRID: AB_1659252
I-A/I-E-APC/Fire™ 750	Biolegend	RRID: AB_2616728
I-A/I-E-BV510	Biolegend	RRID: AB_2561397
IFNγ-PE/Cy7	Biolegend	RRID: AB_2295770
IL-17α-PerCP/Cy5.5	BD Horizon	RRID: AB_2738642
IL-4-FITC	Thermo Fisher Scientific	RRID: AB_465387
Influenza A Virus Nucleoprotein-FITC	Abcam	RRID: AB_445914
SIRPα-PE	Biolegend	RRID: AB_2563549
TCR Vα2-Pe/Cy7	Thermo Fisher Scientific	RRID: AB_2573472
TCRVβ5.1 5.2-PE	Biolegend	RRID: AB_10612761
Anti-mouse IgG, HRP-linked (polyclonal)	Cell Signaling Technology	RRID: AB_330924
Anti-rabbit IgG, HRP-linked (polyclonal)	Cell Signaling Technology	RRID: AB_2099233
Beta-actin (13-E4) (monoclonal)	Cell Signaling Technology	RRID: AB_2223172
ADA (polyclonal)	Abcam	Cat# ab217846
PNP (H-7) (monoclonal)	Santa Cruz Biotechnology	RRID: AB_10845931
MTAP (42-T) (monoclonal)	Santa Cruz Biotechnology	RRID: AB_2147095

**Bacterial and virus strains**		

H3N2 IAV strain (A/X-31)	(Everitt et al., 2012)	N/A

**Chemicals, peptides, and recombinant proteins**		

20X LumiGLO Reagent and 20X Peroxide	Cell Signaling Technology	Cat# 7003
3-(hydroxynitrosoamino)-*L*-alanine (*L*-alanosine)	Cayman Chemicals	Cat# 19545, CAS: 5854-93-3
3-Nitrophenylhydrazine hydrochloride	Sigma-Aldrich	Cat# N21804-5G
4X Laemmli sample buffer	Bio-Rad	Cat# 1610747
5(6)-CFDA, SE.	Thermo Fisher Scientific	Cat# C1157, CAS: 150347-59-4
6-Mercaptopurine monohydrate	Sigma-Aldrich	Cat# 852678-1G-A, CAS: 6112-76-1
Acetic acid, glacial	Thermo Fisher Scientific	Cat# 695092, CAS: 64-09-7
AMP Deaminase inhibitor, Cpd3	Sigma-Aldrich	Cat# 533642
Antimycin A	Sigma-Aldrich	Cat# A867
BSA-Palmitate saturated fatty acid complex (5 mM)	Cayman Chemicals	Cat# 29558
CGS-21680 hydrochloride hydrate	Sigma-Aldrich	Cat# C141, CAS: 124182-57-6
Collagenase D	Sigma-Aldrich	Cat# 11088858001
cOmplete Protease Inhibitor Cocktail	Roche	Cat# 11836170001
D-Glucose-^13^C_6_	Sigma-Aldrich	Cat# 389374, CAS: 110187-42-3
ExtrAvidin-R-Phycoerythrin	Sigma-Aldrich	Cat# E4011
FCCP	Sigma-Aldrich	Cat# C2920
Fixable viability dye eFluor450	Thermo Fisher Scientific	Cat# 65-0863-14
Glutaraldehyde solution	Sigma-Aldrich	Cat# G7651, CAS 111-30-8
Guanine	Sigma-Aldrich	Cat# 51030, CAS: 635-39-2
H-2D(b) Influenza A NP^366-374^ ASNENMETM	NIH Tetramer Facility	N/A
H-2D(b) Influenza A PA^224-233^ SSLENFRAYV	NIH Tetramer Facility	N/A
H-2K(b) chicken OVA^257-264^ SIINFEKL	NIH Tetramer Facility	N/A
Hadacidin	SantaCruz Biotechnology	Cat# sc-490177, CAS: 689-13-4
Hypoxanthine-^13^C_5_,^15^N_4_	Cambridge Isotope Laboratories	Cat# CNLM-7894-0
Inosine	Sigma-Aldrich	Cat# I4625, CAS: 58-63-9
Inosine, ^13^C_5_	Omicron Biochemicals	Cat# NUC-072
Inosine, ^15^N_4_	Cambridge Isotope Laboratories	Cat# NLM-4264-PK
*L*-aspartic acid-^13^C_4_,^15^N_1_	Sigma-Aldrich	Cat# 607835, CAS 202468-27-7
*L*-Glutamine-^13^C_5_,^15^N_2_	Sigma-Aldrich	Cat# 607983
*L*-malic acid-^13^C_4_	Cambridge Isotope Laboratories	Cat# CLM-8065-0
LPS (O111:B4)	Sigma-Aldrich	Cat# LPS25
mFlt3L	Miltenyi Biotec	Cat# 130-097-372
mIL-15	Miltenyi Biotec	Cat# 130-094-072
mIL-2	Miltenyi Biotec	Cat# 130-094-054
Mycophenolic acid	Sigma-Aldrich	Cat# M5255-50MG, CAS: 24280-93-1
N-(3-Dimethylaminopropyl)-N′-ethylcarbodiimide hydrochloride	Sigma-Aldrich	Cat# E6383-5G
Oligomycin	Sigma-Aldrich	Cat# 75371, CAS: 579-13-5
OVA^257-264^ SIINFEKL	Invivogen	Cat# vac-sin
OVA^323-339^ ISQAVHAAHAEINEAGR	Invivogen	Cat# vac-isq
Ovalbumin	Sigma-Aldrich	Cat# A5503
Ovalbumin, Alexa Fluor 488 Conjugate	Thermo Fisher Scientific	Cat# O34781
Ovalbumin, Alexa Fluor 647 Conjugate	Thermo Fisher Scientific	Cat# O34784
Palmitic acid	Sigma-Aldrich	Cat# P0500, CAS 57-10-3
Palmitic acid-^13^C_16_	Sigma-Aldrich	Cat# 605573, CAS: 56599-85-0
Paraformaldehyde	Thermo Fisher Scientific	Cat# 28908, CAS: 3025-89-4
Pierce BCA Protein Assay Kit	Thermo Fisher Scientific	Cat#23225
Poly-L-Lysine	Sigma-Aldrich	Cat# P8920-100ML, CAS: 25988-63-0
Psicofuranine	Cayman Chemicals	Cat# 19574, CAS: 1874-54-0
Pyridine	Sigma-Aldrich	Cat# 270970-100ML
Rotenone	Sigma-Aldrich	Cat# R8875, CAS: 83-79-4
SCH 58261	Sigma-Aldrich	Cat# S4568, CAS: 160098-96-4
Sodium α-ketobutyrate	Sigma-Aldrich	Cat# K0875, CAS 2013-26-5
Sodium α-ketobutyrate-^13^C_4_	Cambridge Isotope Laboratories	Cat# CLM-6164-0.5, CAS: 2483736-24-7
Sodium chloride	Sigma-Aldrich	Cat# S9888, CAS 7647-14-5
Sodium pyruvate	Sigma-Aldrich	Cat# P5280, CAS 113-24-6
Sodium pyruvate-^13^C_3_	Sigma-Aldrich	Cat# 490717, CAS 142014-11-7
SYTOX Blue Dead Cell Stain	Thermo Fisher Scientific	Cat# S34857
SYTOX Green Nucleic Acid Stain	Thermo Fisher Scientific	Cat# S7020
β-lactamase	Sigma-Aldrich	Cat# P0389, CAS: 9073-60-3

**Critical commercial assays**		

Anti-PE MicroBeads	Miltenyi Biotec	Cat# 130-048-80
CD11c MicroBeads UltraPure	Miltenyi Biotec	Cat# 130-108-338
CD4^+^ T Cell Isolation Kit, mouse	Miltenyi Biotec	Cat# 130-104-454
CD8α^+^ T Cell Isolation Kit, mouse	Miltenyi Biotec	Cat# 130-104-075
Foxp3 / Transcription Factor Staining Buffer Set	Thermo Fisher Scientific	Cat# 00-5523-00
IFNγ gamma Mouse ELISA kit	Thermo Fisher Scientific	Cat# 88-7314-22; RRID: AB_2575066
*In situ* Cell Death detection Kit, POD	Sigma-Aldrich	Cat# 11684817910
LEGENDplex Mouse Inflammation Panel	Biolegend	Cat# 740150
LiveBLAzer FRET-B/G Loading Kit with CCF4-AM	Thermo Fisher Scientific	Cat# K1095
Mouse Dendritic Cell Nucleofection Kit	Lonza	Cat# VVPA-1011
Mouse Granzyme B DuoSet ELISA	R&D Systems	Cat# DY1865
Mouse IFN-α ELISA kit (TCM)	PBL Assay Science	Cat# 42120-1
Mouse IL-2 ELISA kit	Thermo Fisher Scientific	Cat# 15530997
pHrodo Red AM Intracellular pH Indicator	Thermo Fisher Scientific	Cat# P35372
Red Blood Cell Lysis Solution (10 × )	Miltenyi Biotec	Cat# 130-094-183
RNeasy Mini Kit	QIAGEN	Cat# 74104
RNeasy Plus Micro Kit	QIAGEN	Cat# 74034
Thermo Fisher Scientific IL-12 p70 Mouse Uncoated ELISA Kit	Thermo Fisher Scientific	Cat# 12384003
TruSeq stranded mRNA library prep kit	Illumina	Cat# 20020594

**Deposited data**		

RNA-Seq (Dendritic cell dataset)	This paper	GEO: GSE126473
RNA-Seq (T cell dataset)	This paper	GEO: GSE147370

**Experimental models: Cell lines**		

LL2-ovalbumin	(Kraman et al., 2010)	N/A
MEFs, bm1 T OVA	(Sancho et al., 2009)	N/A

**Experimental models: Organisms/strains**		

Mouse: *Famin*^−/−^	(Cader et al., 2016)	N/A
Mouse: *Famin*^+/+^	(Cader et al., 2016)	N/A
Mouse: *Famin*^p.254I^	(Cader et al., 2016)	N/A
Mouse: *Famin*^p.254V^	(Cader et al., 2016)	N/A
Mouse: *Famin*^p.284R^	(Cader et al., 2016)	N/A
Mouse: *Famin*^WT^ (*Famin*^fl/fl^)	N/A	N/A
Mouse: *Famin*^ΔDC^ (*Famin*^fl/fl^;*Cd11c*-*Cre*)	N/A	N/A
Mouse: OT-I;*Rag1*^−/−^	(Barnden et al., 1998)	N/A
Mouse: OT-II;*Rag2*^−/−^	(Hogquist et al., 1994)	N/A

**Oligonucleotides**		

ON-TARGETplus Mouse Adsl siRNA	Horizon	Cat# L-064380-01
ON-TARGETplus Mouse Adss siRNA	Horizon	Cat# L-060265-01
ON-TARGETplus Mouse Ampd2 siRNA	Horizon	Cat# L-063716-01
ON-TARGETplus Mouse Ampd3 siRNA	Horizon	Cat# L-042904-01
ON-TARGETplus Mouse Gmpr siRNA	Horizon	Cat# L-046519-01
ON-TARGETplus Mouse Gmps siRNA	Horizon	Cat# L-049796-01
ON-TARGETplus Mouse Impdh1 siRNA	Horizon	Cat# L-042235-01
ON-TARGETplus Mouse Impdh2 siRNA	Horizon	Cat# L-062809-01
ON-TARGETplus Mouse Cpt1 siRNA	Horizon	Cat# L-042456-01
ON-TARGETplus Non-targeting Control Pool	Horizon	Cat# D-001810-10
Murine *Adss* F 5′-CTGGCCACACAGTTGTCGTA-3′; R 5′- AAGCCTTTTCTCCCAGCCAT-3′	Thermo Fisher Scientific	N/A
Murine *Adsl* F 5′-GGATCACCAGAAGGTGGAGC-3′; R 5′-TGTGCACCGATGCTCCTAAG-3′	Thermo Fisher Scientific	N/A
Murine *Ampd2* F 5′-CTCCTTGCATTTGCCATCCAT-3′; R 5′-CCTCTCCGCTACAGTCTGC-3′	Thermo Fisher Scientific	N/A
Murine *Ampd3* F 5′-CTGCGACCGGATCATCTTGAA −3′; R 5′- GTTGGCGGAGAAGGTGTTTG −3′	Thermo Fisher Scientific	N/A
Murine *Cpt1a* F 5′-TGGCATCATCACTGGTGTGTT-3′; R 5′-GTCTAGGGTCCGATTGATCTTTG-3′	Thermo Fisher Scientific	N/A
Murine *Gmpr2* F 5′-CAGCATCCATCAGTGGCAAGAG-3′; R 5′-CCGTTAGCCACATCCAGGCATA-3′	Thermo Fisher Scientific	N/A
Murine *Gmps* F 5′- CCTTGTTGCCAGTGGTAAAGCC −3′; R 5′- TCTTCTGGCAGGTCAAGCTCTC-3′	Thermo Fisher Scientific	N/A
Murine *Impdh1* F 5′- GGCTACGTTCCCGAGGATG −3′; R 5′-GGCTGATGTCAGGTCCACTT-3′	Thermo Fisher Scientific	N/A
Murine *Impdh2* F 5′- CTTGCTGGTGTGGATGTAGTGG −3′; R 5′-GCCTCCAATGACCTGTAGACTG-3′	Thermo Fisher Scientific	N/A
Murine *Famin* F 5′- TGGGGTTGCTCACTCCGGCTG-3′; R 5′-GGAGACTGCTGATTCTTTGGGAAGA-3′	Thermo Fisher Scientific	N/A
Murine *ActinB* F 5′- GATGCTCCCCGGGCTGTATT-3′; R 5′-GGGGTACTTCAGGGTCAGGA-3′	Thermo Fisher Scientific	N/A
Influenza A virus H3N2 A/X-31 M protein F 5′- GGACTGCAGCGTTAGACGCTT-3′; R 5′-CATCCTGTTGTATATGAGGCCCAT-3′	Thermo Fisher Scientific	N/A

**Software and algorithms**		

Adobe Illustrator CC 2019 (23.0.3)	Adobe	https://www.adobe.com/products/illustrator.html; RRID: SCR_010279
Compound Discoverer 2.1 and Compound Discoverer 3.1	Thermo Fisher Scientific	Cat# OPTON-30834
FlowJo	FlowJo LLC	version v10.6.2
GraphPad Prism 8.0	GraphPad Software LLC	version 8.3.0
R 3.6.1	The Comprehensive R Archive Network	Version 3.6.1
Thermo Xcalibur 4.1	Thermo Fisher Scientific	Cat# OPTON-30382; RRID: SCR_014593

**Other**		

Algal lyophilized cells ^13^C (*Synechococcus* sp.)	Sigma-Aldrich	Cat# 487945-1G
Fetal Bovine Serum	Sigma-Aldrich	Cat# F7524-500ML
HBSS	Thermo Fisher Scientific	Cat# 14170112
HEPES	Thermo Fisher Scientific	Cat# 15630049
OptiMEM	Thermo Fisher Scientific	Cat# 31985062
Penicillin-streptomycin	Sigma-Aldrich	Cat# P0781-50ML
RPMI 1640	Thermo Fisher Scientific	Cat# 11875093
RPMI 1640, no glutamine	Thermo Fisher Scientific	Cat# 21870084
RPMI 1640, no glucose	Thermo Fisher Scientific	Cat# 11879020
Seahorse XF base medium	Thermo Fisher Scientific	Cat# 102353-100

### Resource availability

#### Lead contact

Further information and requests for materials should be directed to and will be fulfilled by the Lead Contact, Arthur Kaser (ak729@cam.ac.uk).

#### Materials availability

Unique resources generated in this study are available on reasonable request, although may require completion of a Materials Transfer Agreement.

### Experimental model and subject details

#### Mice

Age-, gender-, and, whenever possible, littermate-matched 6- to 11-week-old mice were used for all experiments. Mice were housed in a 19-21°C environment on a 12 h light/ dark cycle. Health status screening was performed every three months using sentinel mice. Mice were fed on a universal maintenance chow diet purchased from Safe (Safe 105). *Famin*^+/+^, *Famin*^−/−^, *Famin*^p.254I^, *Famin*^p.254V^ and *Famin*^p.284R^ mice, generated on a C57BL/6NTac background, have previously been described (Cader et al., 2016). The loxP-flanked conditional allele (*Famin*^fl/fl^) allele was generated by converting mice with homologous recombination of the tm1a(KOMP)Wtsi construct (Cader et al., 2016) with a FlpO recombinase-transgenic mouse. *Famin*^ΔDC^ mice were then generated by crossing *Famin*^fl/fl^ mice with *Itgax*-Cre (CD11c-Cre) mice (B6.Cg-Tg(Itgax-cre)1-1Reiz/J, on a C57BL/6 background). *Itgax*-Cre;*Famin*^fl/fl^ mice and their controls were born at Mendelian ratio and developed normally, consistent with what we previously reported for the other *Famin* mutant mice used in this study (Cader et al., 2016). None of these mice develop spontaneous autoimmunity or autoinflammation under specific pathogen-free conditions. OT-I;*Rag*^−/−^ (‘OT-I’) and OT-II;*Ptprc*^*a*^;*Rag2*^−/−^ (‘OT-II’) mice on a C57BL/6 background have previously been described (Barnden et al., 1998; Hogquist et al., 1994). Maintenance and breeding under specific pathogen-free conditions was performed at the Central Biomedical Services facility or Phenomics Laboratory, University of Cambridge. UK Home Office and local ethics approval has been granted for all experimental procedures.

#### Dendritic cell and T cell isolation

DCs were isolated from spleens digested in 1 mg/mL collagenase D (Sigma, 11088858001) in complete media in the presence of DNase (Sigma, D4263). Positive selection for CD11c^+^ DCs was performed using CD11c Ultrapure beads following the manufacturer’s protocol (Miltenyi, 130-100-875). BM-derived DCs were isolated from murine tibias and femurs by flushing in complete RPMI-1640 medium, filtering through a 70 μm cell strainer, lysing red blood cells using red blood cell lysis solution (Miltenyi, 130-094-183) in accordance with manufacturer’s instructions, and subsequent resuspension in complete RPMI-1640 medium (containing 100 U/mL of penicillin-streptomycin, 10 mM HEPES buffer and 10% FBS) followed by culture for 9 days in mFlt3L (100 ng/mL, Miltenyi, 130-097-372), replenished on days 3 and 6. After 9 days in culture, isolation of BM-derived DCs into cDC1 and cDC2 subsets was performed using a protocol adapted from (Zelenay et al., 2012). Briefly, 9-day Flt3L-expanded BM-derived cDC2s were isolated by positive selection using SIRPα-PE antibody (Biolegend, 144011) and anti-PE microbeads (Miltenyi, 130-048-80), followed by negative selection of cDC1 through depletion of plasmacytoid DCs with anti-B220-PE antibody (Biolegend, 103207) and anti-PE microbeads (Miltenyi, 130-048-80). For maturation prior to all subsequent analysis, splenic DCs were rested overnight, while BM-derived cDC1s or cDC2s were treated with 1 μg/mL LPS (O111:B4, Sigma, LPS25) for 18 h. All treatments of splenic DCs or BM-derived cDC1s were performed during this maturation step unless specified otherwise. Inhibitors were used at the following final concentrations: 25 μM (Figures 4A and S4A) or 100 μM (Figure 5C) *L*-alanosine (Cayman Chemicals,19545), 50 μM hadacidin (SantaCruz, sc-490177), 5 μM 6-mercaptopurine (Sigma, 852678-1G-A), 5 μM Cpd3 (Sigma, 533642), 0.8 (Figures 4H and 5B) or 1 μM (Figures 5D, 5E, and 7F) mycophenolic acid (Sigma, M5255-50MG), 100 μM psicofuranine (Cayman Chemicals, 19574). Guanine was used at 100 μM (Sigma, 51030); pyruvate (Sigma, P5280) and α-ketobutyrate (Sigma, K0875) were used at 1 mM.

T cells isolated from spleens and lymph nodes of OT-I;*Rag*^−/−^ and OT-II;*Ptprc*^*a*^;*Rag2*^−/−^ mice were purified using negative selection for CD8^+^ or CD4^+^ T cells (Miltenyi, 130-104-075 and 130-104-454). Purity (80%–90%) was confirmed by flow cytometry.

Either male or female mice were taken for DC/ T cell isolation – each individual experiment was gender-matched. All cells were cultured at 37°C, 5% CO_2_.

### Method details

#### Influenza infection

Isoflurane anesthetized 6-8 week-old female mice were infected by intranasal inoculation with 10^4^ plaque forming units (PFU) Influenza A virus H3N2 A/X-31 strain in 50 μL of sterile PBS. Disease activity was scored in a blinded to genotype and group allocation, using the following scoring system: 5 ‒ healthy mouse; 4 ‒ mouse is huddled, looks hypothermic; 3 ‒ ruffled fur, squinting eyes; 2 ‒ mouse is hunched; 1 ‒ mouse looks lethargic. LEGENDplex Mouse Inflammation Panel (Biolegend, 740150) was used to determine cytokine levels in plasma according to manufacturer’s protocol and fluorescent signals assessed by flow cytometry. Absolute quantification was performed using standard curves generated in the assay. For analysis of influenza-specific T cells bronchoalveolar lavage with cold PBS was performed. Influenza viral load was determined in total RNA extracted from lungs (RNeasy kit, QIAGEN, 74104), via qRT-PCR using primers for influenza M protein transcript, 5′-GGACTGCAGCGTTAGACGCTT-3′; and 5′-CATCCTGTTGTATATGAGGCCCAT-3′ (Prabhu et al., 2013). Influenza M protein mRNA levels were normalized to m*Actb* (5′-GATGCTCCCCGGGCTGTATT-3′ and 5′-GGGGTACTTCAGGGTCAGGA-3′). Terminal deoxynucleotidyl transferase (TdT) dUTP nick-end labeling (TUNEL) was performed on formalin-fixed paraffin embedded sections of lungs using the *In Situ* Cell Death Detection Kit, POD (Roche, 11684817910) following manufacturer’s instructions. An Olympus CX31 microscope with Nikon DS-Fi2 camera attachment was used to analyze the sections.

#### *In vitro* T cell priming and restimulation

Splenic DCs were rested overnight in normal media, and BM-derived cDC1 or cDC2s were activated with 1 μg/mL LPS (O111:B4, Sigma, LPS25) for 18 h prior to pulsing with antigen. Mature DCs were pulsed for 30 min with 1 μg/mL (splenic CD11c^+^ DCs) or 0.5 μg/mL (cDC1 and cDC2 BM-derived DCs) OVA^257-264^ peptide (SIINFEKL) (Invivogen, vac-sin), 1 μg/mL OVA^323-339^ peptide (ISQAVHAAHAEINEAGR) (Invivogen, vac-isq), 1 mg/mL of ovalbumin (Sigma, A5503), or UV-irradiated bm1T-OVA MEFs (Sancho et al., 2009) as indicated. OT-I and OT-II cells were labeled with carboxyfluorescein succinimidyl ester (CFSE; Molecular Probes, C1157) according to manufacturer’s protocol prior to co-culture with antigen-pulsed DCs. OT-I cells and DCs were co-cultured for 72 h at a ratio of 5:1 for cDC1 and splenic DCs; OT-II cells and DCs were co-cultured at 2:1 for 96 h. For differentiation into T_E_ and T_EM_ cells 72 h after priming, OT-I cells were passaged for 5 days with mIL-2 (5,000 IU/mL, Miltenyi, 130-094-055) or mIL-15 (100 IU/mL, Miltenyi, 130-094-072), respectively (Manjunath et al., 2001). Primed OT-I cells were re-seeded at the indicated time-points at 10^6^ cells/mL, restimulated for 5 h with plate-bound anti-CD3 (5 μg/mL, Thermo Fisher Scientific, 16-0037-81) and soluble anti-CD28 (2 μg/mL, Thermo Fisher Scientific, 16-0289-81), and supernatants were then analyzed for IFNγ release. For analysis of intracellular cytokines, OT-II cells were rested for 3 days following 96 h co-culture with DCs prior to restimulation for 5 h with 1 μg/mL OVA^323-339^ or anti-CD3/CD28. For co-culture experiments using fixed dendritic cells, mature splenic DCs were pre-loaded with 1 μg/mL of OVA^257-264^ for 30 min and fixed for 10 min in 0.01 or 0.05% of glutaraldehyde solution (Sigma, G7651). Following 2 washes in PBS, CFSE-labeled OT-I T cells were added at 5:1 ratio and co-cultured for 72 h.

#### Cytotoxicity assays

Cytotoxicity was measured as previously described (Noto et al., 2013). Briefly, after 72 h of priming, OT-I T cells were passaged twice at 48 h intervals in media containing 5,000 IU/mL mIL-2. Effector T cells were then combined with target cells at the indicated ratios for 5 h at 37°C. Target cells were splenocytes from wild-type mice pulsed with 1 μg/mL of OVA^257-264^ for 30 min and labeled with 5 μM of CFSE (CFSE_high_), which were combined at 1:1 ratio with control splenocytes labeled with 0.5 μM CFSE (CFSE_low_). Specific lysis was equal to 100 – ((CFSE_high_/CFSE_low_) in the presence of cytotoxic CD8 T cells / (CFSE_high_/CFSE_low_) in the absence of cytotoxic CD8 T cells) × 100. Supernatants were harvested for cytokine analysis by ELISA.

#### *In vivo* T cell priming

CD8^+^ T cells and CD4^+^ T cells were isolated from spleens and lymph nodes of OT-I;*Rag*^−/−^ and OT-II;*Ptprc*^*a*^*;Rag2*^−/−^ mice, respectively. Following labeling with 5 μM of CFSE, 5 × 10^6^ T cells/mouse were intravenously injected into *Famin*^ΔDC^ mice and their controls. 24 h later, mice were immunized with 25 μg ovalbumin intraperitoneally. Three days later, proliferation indices of splenic T cells were calculated based on CFSE dilution and, in the case of OT-I cells, CTL activity of total splenocytes analyzed as described above.

#### Flow cytometry

Cells were blocked with purified anti-CD16/CD32 antibody (Biolegend, 101302) for 30 min on ice and stained with corresponding antibodies for cell surface molecules. For intracellular staining, samples were fixed with 2% paraformaldehyde (Thermo Fisher Scientific, 28908), permeabilized using Wash/Perm buffer (BD Biosciences, 554723), and stained for intracellular cytokines using standard protocols; FOXP3 was stained with Mouse *Foxp3* buffer set (Biolegend, 560409) per manufacturer’s protocol. Peptide-tetramer staining was analyzed in whole blood obtained through tail bleeds, with antibodies and peptide-loaded tetramers added directly to blood, and samples diluted with buffer prior to analysis by flow cytometry. Monomers for H-2D^b^ Influenza A NP^366-374^ (ASNENMETM), H-2D^b^ Influenza A PA^224-233^ (SSLENFRAYV), and H-2K^b^ OVA^257-264^ (SIINFEKL) were obtained from the NIH Tetramer Core Facility and tetramers prepared using Extravidin–PE (Sigma, E4011) as per standard protocol. Single cell fluorescence was analyzed on BD LSRFortessa or Attune NxT flow cytometers. Data analysis was performed using FlowJo software v9/v10; gating strategies for experiments are listed in Table S7.

#### Cytokine measurement by ELISA

Supernatants from experiments were analyzed using ELISA according to the manufacturer’s instructions (IFNγ, Thermo Fisher Scientific, 15501107; IFN-α, PBL Assay Science, 42120-2; IL-2, Thermo Fisher Scientific, 15133787; IL-12p70, Thermo Fisher Scientific, 12384003; granzyme B, R&D Systems, DY1865).

#### RNA extraction and sequencing

RNeasy Mini Kit (QIAGEN, 74104) was used to extract RNA and samples were quantified with a NanoDrop ND-1000 spectrophotometer (Thermo Fisher Scientific). RNA quality was assessed using the Agilent 2200 or 2100 TapeStation system (Agilent Technologies). Libraries were prepared using TruSeq stranded mRNA library prep kit (Illumina, 20020594) in accordance with the manufacturer’s instructions. Sequencing of libraries was performed using an Illumina NextSeq 500 platform with NextSeq 500-Mid Output kit generating 1x75 bp end reads (T cell dataset GSE147370) or 2x150bp end reads (dendritic cell dataset GSE126473). FastQ files were quality-checked (FastQC; http://www.bioinformatics.babraham.ac.uk/projects/fastqc/) and any residual adaptor sequences were removed (TrimGalore; http://www.bioinformatics.babraham.ac.uk/projects/trim_galore/). Reads were subsequently aligned to the appropriate reference genome (mm10, UCSC for cDCs, Ensembl Mus_musculus.GRCm38 for T cells) using HISAT2 (Kim et al., 2015) (for cDCs) or STAR (Dobin and Gingeras, 2015) (for T cells). Analysis of differentially expressed genes was conducted on read count files using the limma package in R with the Voom transformation (for cDCs) or edgeR (for T cells). Gene Set Enrichment Analysis (GSEA) was undertaken using log counts-per-million (CPM) data. RNA-Seq data generated as part of this study can be accessed at the Gene Expression Omnibus (GEO: GSE126473, GSE147370).

#### Immunoblot

6 × 10^6^ BM-derived cDC1s were washed once in ice-cold PBS and lysed in ice-cold RIPA buffer supplemented with protease inhibitors (cOmplete Protease Inhibitor Cocktail, Roche). After lysing for 15 min on ice, cell debris was removed by centrifugation for 15 min at 4°C. Protein levels were quantified using the Pierce BCA Protein Assay Kit (Thermo Scientific) and samples were normalized to protein content before addition of 4X laemmli buffer (Bio-Rad) and boiling at 95°C for 5 min. Samples were run on a 10% SDS-PAGE gel. Proteins were transferred to a nitrocellulose membrane using a Trans-Blot Turbo transfer system before blocking for 1 h at room temperature in 5% milk in TBS-T. Membranes were incubated with primary antibodies overnight at 4°C in 5% milk in TBS-T. These were detected by incubation with HRP-conjugated secondary antibodies for 1 h at room temperature and visualized using 20X LumiGlo reagent (Cell Signaling).

#### Metabolic tracing experiments

For metabolic tracing experiments using BM-derived cDC1s, cells were isolated and stimulated for 18 h with 1 μg/mL LPS as described above. Following this, cells were incubated for 3 h with 300 μM [^13^C_4_] malic acid (Cambridge Isotope Laboratories, CLM-8065-0), 100 μM [^13^C_16_] palmitic acid (Sigma-Aldrich, P0500), 1 mM [^13^C_3_] sodium pyruvate (Sigma-Aldrich, 490717), 1 mM [^13^C_4_] α-ketobutyrate (Cambridge Isotope Laboratories, CLM-6164) or 150 μM *L*-aspartic acid-^13^C_4_,^15^N_1_ (Sigma-Aldrich, 607835) supplemented into complete RPMI-1640 medium as applicable. In glutamine labeling experiments, BM-derived cDC1s were incubated for 3 h with 2 mM [^15^N_2_^13^C_5_] glutamine (Sigma-Aldrich, 607983), added into RPMI glutamine free medium. For glucose labeling experiments, BM-derived cDC1s were pulsed for 1 h with 2 g/L [^13^C_6_] glucose (Sigma-Aldrich, 389374) supplemented into RPMI glucose free medium. For metabolic tracing experiments utilizing splenic CD11c^+^ DCs, cells were isolated and rested overnight before pre-equilibration in OptiMEM for 3 h and subsequent addition of [^13^C_5_^15^N_4_] hypoxanthine (Cambridge Isotope Laboratories, CNLM-7894-0) to a final concentration of 25 μM for 3 h prior to harvesting of supernatants and cell extracts.

For measurements of systemic inosine levels plasma was collected by cardiac puncture followed by centrifugation in EDTA-coated tubes for 15 min at 4°C at 2000 g. 20 μL aliquots were taken and prepared for LC-MS by addition of 100 uL 4:1 methanol:water followed by vortexing and centrifugation at 20 000 g. The supernatants were then dried using a centrifugal evaporator (Savant, ThermoFisher). Samples were reconstituted in 100 μL ammonium acetate containing 2 μM [^13^C_10_, ^15^N_5_] adenosine monophosphate and adenosine triphosphate, 10 μM [^13^C_4_] succinic acid, a 1 in 5000 diluted [U^13^C, U^15^N] mixture of amino acids (all purchased from Sigma Aldrich) and 50 nM [^13^C_5_] inosine (Cambridge Isotope Laboratories) as internal standards. *Famin*^p.254I^, *Famin*^p.254V^ and *Famin*^p.284R^ mice were fasted for 18 h prior to harvesting.

#### Extraction of aqueous metabolites

After washing with PBS or 162 mM ammonium acetate adjusted to pH 7.4 (as appropriate), cell pellets were then extracted using the 2:1 chloroform:methanol method described by Folch (Folch et al., 1957) with modifications to the method as previously detailed (Cader et al., 2020). All solvents used were HPLC or LC-MS grade and obtained from Fisher Scientific. Aqueous extracts were stored at −80°C prior to analysis.

#### LC-MS sample preparation

Aqueous extracts of cells were dried using a centrifugal evaporator (Savant, ThermoFisher) and reconstituted in 10 mM ammonium acetate containing 2 μM [^13^C_10_, ^15^N_5_] adenosine monophosphate and adenosine triphosphate, 10 μM [^13^C_4_] succinic acid, and a 1 in 5000 diluted [U^13^C, U^15^N] mixture of amino acids (all purchased from Sigma Aldrich) as internal standards. Where appropriate, internal standards were omitted during isotopic labeling experiments to prevent contamination with labeled substrates. The samples were then vortexed and sonicated for 5 min, followed by brief pulsed centrifugation to recover maximum volume.

Molecular formula determination using accurate mass and isotopic mass distribution, confirmed by authentic standard, were used to validate identification of inosine as the top-ranking identifiable LC-MS feature of differential abundance between supernatants of *Famin*^+/+^ and *Famin*^−/−^ splenic DCs. For analysis of cell culture supernatants in subsequent experiments, 20 μl of supernatant was aliquoted directly onto a styrene 96 well plate (Corning) followed by dilution with 100 μl of 10 mM ammonium acetate containing 50 nM [^13^C_5_] inosine (Omicron Biochemicals) or 50 nM [^15^N_4_] inosine (Cambridge Isotope Laboratories) as an internal standard. Where appropriate the internal standard was omitted. For absolute quantitation of both labeled and unlabelled inosine, an inosine calibration line was prepared in the appropriate cell culture medium in the following concentrations: 100 pM, 1 nM, 5 nM, 10 nM, 50 nM, 100 nM and 1 μM. These calibrants were then subjected to the same dilution and preparation described above. All plates were sealed with a pre-slit silicone sealing mat prior to injection (Thermo Fisher Scientific).

#### LC-MS analysis of aqueous metabolites

A Q Exactive Plus orbitrap coupled to a Vanquish Horizon ultra high performance liquid chromatography system was used for all the analysis. LC-MS methodology used corresponds to the ACE C18-PFP and the Phenomenex Gemini-NX protocols described previously (Cader et al., 2020), utilizing identical chromatographic and MS parameters. The majority of analyses (for example detection of nucleotides, nucleosides and organic acids etc.) was carried out using the ACE C18-PFP column and, where appropriate, nucleoside phosphates were measured on a BEH amide HILIC column as detailed in Cader et al. (2020). For analysis of supernatants, where sensitivity was critical, 10 μL was injected with the first minute of chromatography being switched to waste to prevent build-up of matrix containing contaminants in the source of the mass spectrometer.

All solvents and additives used were LC-MS or Optima grade and obtained from Fisher Scientific or Merck.

#### Hydrazone derivatization of keto acids and hydroxy carboxylic acids in cell culture supernatants and subsequent LC-MS analysis

An internal standard solution was prepared by extracting 100 mg of U^13^C lyophilized algae (Sigma) using the Folch extraction described above. Supernatants were dried using a centrifugal evaporator (Savant, Thermo Fisher) and derivatised according to a modified version of the protocol previously described (Han et al., 2013). Briefly, 50 μl of 75% aqueous methanol was added to the dried culture medium followed by 10 μl of the internal standard mix. To this mixture, 30 μl of 250 mM 3-nitrophenylhydrazine (in 50% aqueous methanol), 30 μl of 150 mM 1-ethyl-3-(3-dimethylaminopropyl)carbodiimide (in methanol) and 30 μl of 7.5% pyridine (in 75% aqueous methanol) were added sequentially. The resulting mixture was vortexed and samples allowed to derivatise for 1 h on ice. Samples were subsequently quenched with 5 mg/mL butylated hydroxytoluene and 420 μl of water and centrifuged to pellet any salts from the media.

The LC gradient employed for the separation of the hydrazone derivatives utilized a binary solvent mixture consisting of mobile phase A, 0.1% formic acid in water and B, 0.1% formic acid in methanol and the column was an Acquity CSH C18 (100 × 2.1mm, 1.7 μm). The gradient program was as follows: 18% B was increased to 90% B in a linear gradient over 6.75 min, held at 90% for a further minute followed by re-equilibration for 1 min to give a total run time of 9 min. The flow rate was 400 μl/min and the column oven temperature was 40°C. The injection volume was 5 μl. To prevent derivatisation reagents from entering the ion source, a switch was employed for the first 2 min of the gradient program. Samples were run in negative ion mode using MS parameters previously described (Cader et al., 2020).

#### LC-MS data processing

All data were acquired using Xcalibur (Version 4.1, Thermo Fisher Scientific). Targeted processing was carried out using Xcalibur and unbiased analysis using Compound Discoverer (Version 2.1 or Version 3.1, Thermo Fisher Scientific). Untargeted analysis utilized data from both positive and negative ionization modes. Chromatogram peaks for each differential metabolite were manually verified using XCalibur (Version 4.1, Thermo Fisher Scientific) and identities validated using the high-resolution *m/z* METLIN database (Scripps Research Institute). To confirm identification of inosine and in cases of ambiguity, compound retention times were validated against known external standard solutions. For all cellular and serum metabolite analysis, target peak areas corresponding to metabolites were normalized to total ion content unless otherwise indicated. For absolute quantitation of inosine in supernatants, normalization of target peaks was performed with reference to internal standards, and quantitation performed with reference to a calibration line between 10 pM and 1 μM prepared in the appropriate sample matrix. Relative quantitation of metabolite levels in supernatant tracing studies were not normalized.

All sample data were processed using Compound Discoverer (Version 2.1 and Version 3.1, Thermo Fisher Scientific) to accurately calculate total ion content for use as a normalization factor. For labeling studies, incorporation into specific compounds was determined by accurate mass shift of +1.0034 and +0.9970 for ^13^C and ^15^N respectively. Endogenous levels of ^13^C and ^15^N compounds of interest were determined by reference to control samples pulsed with unlabelled compounds of investigation, and endogenous levels subtracted from quantified isotopomers in the labeled samples as applicable.

#### Determination of extracellular acidification rate and oxygen consumption rate

Bone marrow-derived cDC1 stimulated overnight with LPS (O111:B4, Sigma-Aldrich, LPS25), or isolated splenic DCs matured overnight, were seeded prior to analysis at 3 × 10^5^ cells per well on Poly-L-lysine-coated plates (Sigma-Aldrich, P8920), as indicated. Cells were then washed twice and incubated for 1 h in XF assay medium (unbuffered DMEM pH 7.4 with 10 mM glucose, 100 μM sodium palmitate and 2 mM L-glutamine) in a non-CO_2_ incubator at 37°C as per manufacturer’s instructions (Agilent Technologies). Measurements of extracellular acidification rate (ECAR) and oxygen consumption rate (OCR) were determined using an XF-96 Extracellular Flux Analyzer (Agilent Technologies). Serial measurements were obtained under basal conditions and following addition of 1 μM oligomycin (Sigma-Aldrich, 75371), 1.5 μM FCCP (Sigma-Aldrich, C2920) and 100 nM rotenone with 1 μM antimycin A (Sigma-Aldrich, R8875 and A867). For determination of glycolysis, ECAR measurements were obtained under basal conditions.

#### Cytoplasmic pH assay

Intracellular pH was compared using pHrodo Red AM (Thermo Fisher Scientific, P35372) fluorogenic probe for measurement of cytoplasmic pH according to manufacturer’s protocol. In brief, splenic DCs were matured overnight and incubated with 5 μM pHrodo for 30 min at 37°C in a non-CO_2_ incubator in Hank’s Balanced Salt Solution (HBSS) and washed once before fluorescence signal was measured using a microplate reader (Tecan infinite M1000, Tecan Group or CLARIOstar plus, BMG Labtech) with an excitation/emission of 560/580nm.

#### Antigen uptake assay

LPS-treated cDC1s or splenic DCs were incubated with 0.05 mg/mL of OVA-AF647 (Thermo Fisher Scientific O34784) in HBSS containing HEPES at 37°C in a non-CO_2_ incubator for 30 min, while control cells were kept on ice to account for passive diffusion. Following washes in ice cold HBSS, samples were prepared for analysis by flow cytometry as described above. For analysis of splenic cDC1s and cDC2s cells were gated on CD11c^+^MHC II^+^CD8^+^CD11b^−^ and CD11c^+^MHC II^+^CD11b^+^CD64^−^, respectively.

#### Endosome-to-cytosol uptake assay

Assay was performed as previously described (Cebrian et al., 2011), with some modifications using the LiveBLAzer FRET-B/G Loading Kit with CCF4-AM (Thermo Fisher Scientific, K1095). LPS-treated BM-derived DCs were loaded with 1 μM of CCF4-AM at room temperature, followed by incubation with 2 mg/mL β-lactamase (Sigma, P0389) for the indicated time. Cells were subsequently analyzed by flow cytometry, and blue-to-green (excitation 405 nm, emission 450 nm/525 nm). FRET ratio was used as an indicator for efficiency of antigen export into the cytosol. Response ratios were calculated as per manufacturer’s instructions and normalized to the signal intensity of control cells, which had been incubated on ice.

#### MHC I recycling assay

The rate of MHC I recycling was assessed as previously described (Belabed et al., 2020). LPS-treated cDC1s were incubated in the presence of 1 mM sodium α-ketobutyrate (Sigma-Aldrich, K0875), or control for 18 h. Cells were blocked with anti-CD16/CD32 antibody (Biolegend, 101302) and subsequently incubated with anti-H-2K^b^-FITC (Biolegend, 114605) for 30 min on ice. To enable internalisation, cells were incubated for 30 min at 37°C in complete RPMI-1640 medium with 1 mM sodium α-ketobutyrate replenished as applicable. After washing in 1% BSA-PBS, cells were subsequently incubated in stripping buffer (0.5 M NaCl, 0.5% acetic acid, pH 3.0) for 10 min on ice. After washing with ice cold PBS, cells were fixed in 2% paraformaldehyde (to determine basal MHC I staining after internalisation step) or re-incubated in complete pre-warmed RPMI-1640 medium (with 1 mM sodium α-ketobutyrate replenished if applicable) for 15 min or 30 min to allow MHC I recycling. After re-incubation, cells were incubated in stripping buffer for 10 min on ice, washed in ice cold PBS and fixed in 2% paraformaldehyde followed by analysis by flow cytometry. The difference in mean fluorescence intensity after re-incubation was determined to calculate the % MHC I recycled at each time point as described previously.

#### siRNA transfection

Freshly isolated splenic CD11c^+^ DCs were transfected with 30 pmol/sample of siRNA (purchased from Dharmacon, Horizon Discovery) using a Mouse Dendritic Cell Nucleofector Kit (Lonza, VVPA-1011) and Nucleofector 2b. Cells were rested for 36-48 h prior to assays (antigen uptake or co-culture). RNA for knockdown validation was extracted using RNeasy Plus Micro Kit (QIAGEN), reverse transcribed using M-MLV Reverse Transcriptase (Thermo Fisher) and SYBR Green Q-PCR (Eurogentec) was performed using QuantStudio 7 Flex (Thermo Fisher). For primer sequences see Key resources table.

#### T cell activation assays in presence of DC-secreted soluble factor

Splenic CD11c^+^ DCs were rested overnight in RPMI, and on the next day cultured in OptiMEM or RPMI for 3 h. Cell-free supernatants were harvested and frozen immediately or centrifuged using 3 kDa cut-off spin columns. OT-I or OT-II T cells isolated from spleens and LNs were seeded at 10^5^ cells/mL and stimulated for 72 h with plate-bound anti-CD3 (5 μg/mL, 16-0037-81, Thermo Fisher Scientific) and soluble anti-CD28 (2 μg/mL, 16-0289-81, Thermo Fisher Scientific) in presence of supernatants harvested from DCs, soluble inosine with or without 100 nM SCH 58261 (Sigma Aldrich, S4568) or 0.5 μM CGS-21680 (Sigma Aldrich, C141). For T cell RNA-seq experiments, naive OT-I T cells were cultured in the presence of 2 h splenic DC supernatant and stimulated for 24 h with anti-CD3/CD28 prior to RNA extraction.

#### Tumor xenograft model

2.5 × 10^4^ LL2-OVA cells (for experiments comparing *Famin*^p.254I^, *Famin*^p.254V^, *Famin*^p.284R^ mice) or 2 × 10^4^ LL2-OVA cells (for experiment comparing *Famin*^p.254V^ and *Famin*^p.284R^ mice) in PBS were subcutaneously injected into the left flank of gender- and age-matched mice of 6-10 weeks of age. Tumor growth was assessed at least every other day in a fully blinded fashion, using a calliper for both the long (L) and short (S) dimensions, and tumor volume calculated using the equation volume = (L × S^2^)/2.

### Quantification and statistical analysis

Statistical analyses were performed using Graphpad Prism 6.0 /8 /9 or, and as described in LC-MS analysis methods, Compound Discoverer 2.1 / Compound Discoverer 3.1 (Thermo Scientific). Unless otherwise stated, statistical significance was calculated as appropriate using unpaired, two-tailed Student’s t test or ordinary one-way ANOVA and Tukey post hoc test as described in the figure legends. Formal statistical determination of whether the data met assumptions of the approach was not undertaken. Grubbs’ test was used to identify outliers within datasets. Where indicated, FDR-adjusted p values were calculated using the Benjamini-Hochberg procedure. All *in vivo* experiments were performed in a blinded manner. Data are represented as mean and standard error of the mean (SEM). A *P* value of < 0.05 was considered significant.

## Data Availability

• RNA Sequencing datasets generated in this study have been deposited at the Gene Expression Omnibus (GSE126473 and GSE147370) and are publicly available as of the date of publication. • This paper does not report original code. • Any additional information required to reanalyse the data reported in this paper is available from the lead contact upon reasonable request.

## Supplemental information

Document S1. Figures S1–S7 and Tables S1–S7

Document S2. Article plus supplemental information
