# Supplementary material for: A purine metabolic checkpoint that prevents autoimmunity and autoinflammation
Source: Cell Metab. 2022 Jan 4;34(1):106–124.e10. doi: 10.1016/j.cmet.2021.12.009 (PMC8730334; doi:10.1016/j.cmet.2021.12.009)
Supplement: Document S1. Figures S1–S7 and Tables S1–S7 [file mmc1.pdf]

Cell Metabolism, Volume *34*

Supplemental information

A purine metabolic checkpoint that prevents autoimmunity and autoinflammation

Svetlana Saveljeva, Gavin W. Sewell, Katharina Ramshorn, M. Zaeem Cader, James A. West, Simon Clare, Lea-Maxie Haag, Rodrigo Pereira de Almeida Rodrigues, Lukas W. Unger, Ana Belén Iglesias-Romero, Lorraine M. Holland, Christophe Bourges, Muhammad N. Md-Ibrahim, James O. Jones, Richard S. Blumberg, James C. Lee, Nicole C. Kaneider, Trevor D. Lawley, Allan Bradley, Gordon Dougan, and Arthur Kaser

Figure S1. Compromised FAMIN in DCs augments T cell responses to influenza A virus. Related to Figure 1.

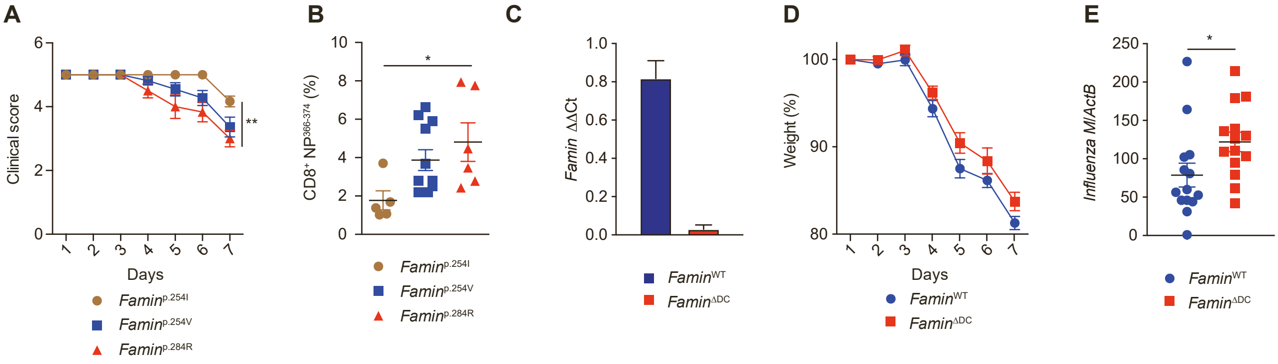

Figure S1. Compromised FAMIN in DCs augments T cell responses to influenza A virus. Related to Figure 1.

(A) Clinical scores of *Famin*$^{p.254I}$, *Famin*$^{p.254V}$, *Famin*$^{p.284R}$ mice during the course of infection with 10^4 plaque-forming units (PFU) of influenza A H3N2 virus, strain A/X-31 (*n* = 6/11/6).

(B) Percentage of $CD3^+$ $CD8^+$ $NP^{366\text{-}374}$ $tetramer^+$ cells in BAL of *Famin*$^{p.254I}$, *Famin*^{p254V}, *Famin*$^{p.284R}$ mice on day 7 post-infection (*n* = 6/11/6).

(C) mRNA levels of *Famin*, expressed as ΔΔCt, from splenic $CD11c^+$ DCs isolated from *Famin*^{WT} or *Famin*$^{\Delta DC}$ mice (*n* = 3).

(D) Percentage weight loss of *Famin*^{WT} or *Famin*$^{\Delta DC}$ mice during the course of infection with 10^4 plaque-forming units (PFU) of influenza A H3N2 virus, strain A/X-31 (*n* = 14/16, two *Famin*$^{\Delta DC}$ mice were sacrificed on day 6 due to excessive weight loss).

(E) Influenza A virus M protein gene expression, expressed as relative ratio to *Actb*, in lung tissue *Famin*^{WT} or *Famin*$^{\Delta DC}$ mice 7 days after infection (*n* = 14/14).

Data represented as mean ± S.E.M. *$p < 0.05$, **$p < 0.01$, and ***$p < 0.001$ (repeated measures (RM) one-way ANOVA, one-way ANOVA or unpaired, two-tailed Student's t test where appropriate).

Figure S2. FAMIN activity in DCs restrains CD4⁺ and CD8⁺ T cell responses. Related to Figure 2.

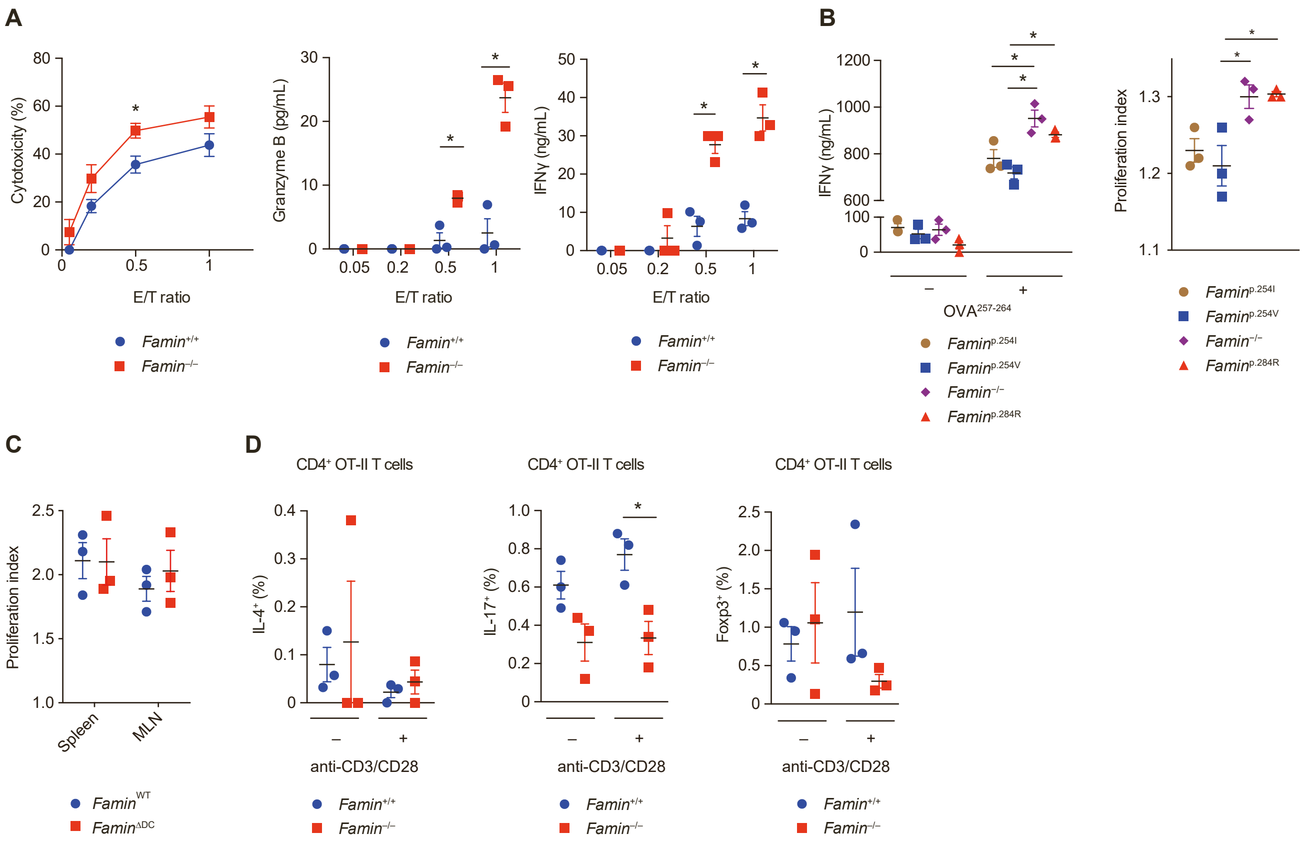

Figure S2. FAMIN activity in DCs restrains CD4$^+$ and CD8$^+$ T cell responses. Related to Figure 2.

(A) Specific cytotoxicity of OT-I T cells primed with OVA$^{257\text{-}264}$-pulsed BM-derived cDC1s from *Famin*$^{-/-}$ and *Famin*$^{+/+}$ mice, assayed against OVA$^{257\text{-}264}$-pulsed wild-type splenocytes at indicated E to T cell ratios, granzyme B and IFNγ secretion during cytotoxicity assays (n = 3).

(B) IFNγ levels and proliferation indices of OT-I T cells following priming with *Famin*$^{p.254I}$, *Famin*$^{p.254V}$, *Famin*$^{p.284R}$, and *Famin*$^{-/-}$ BM-derived cDC1 pulsed with 0.5 μg/ml OVA$^{257\text{-}264}$, measured by CFSE-dilution (n = 3).

(C) Proliferation indices of OT-I T cells in spleen and mesenteric lymph nodes (MLN) after adoptive transfer into *Famin*^{WT} or *Famin*$^{\Delta DC}$ mice, 72 h after immunisation with 25 μg of ovalbumin, measured by CFSE-dilution (n = 3).

(D) Percentage IL-4$^+$, IL-17$^+$ and Foxp3$^+$ OT-II T cells after restimulation with anti-CD3/CD28, following priming with 1 μg/ml OVA$^{323\text{-}339}$-pulsed BM-derived cDC2 7 days earlier (n = 3).

Data represented as mean ± S.E.M. *$p < 0.05$, **$p < 0.01$, and ***$p < 0.001$ (one-way ANOVA or unpaired, two-tailed Student's t test where appropriate).

Figure S3. FAMIN activity in DCs biochemically controls antigen uptake and presentation. Related to Figure 3.

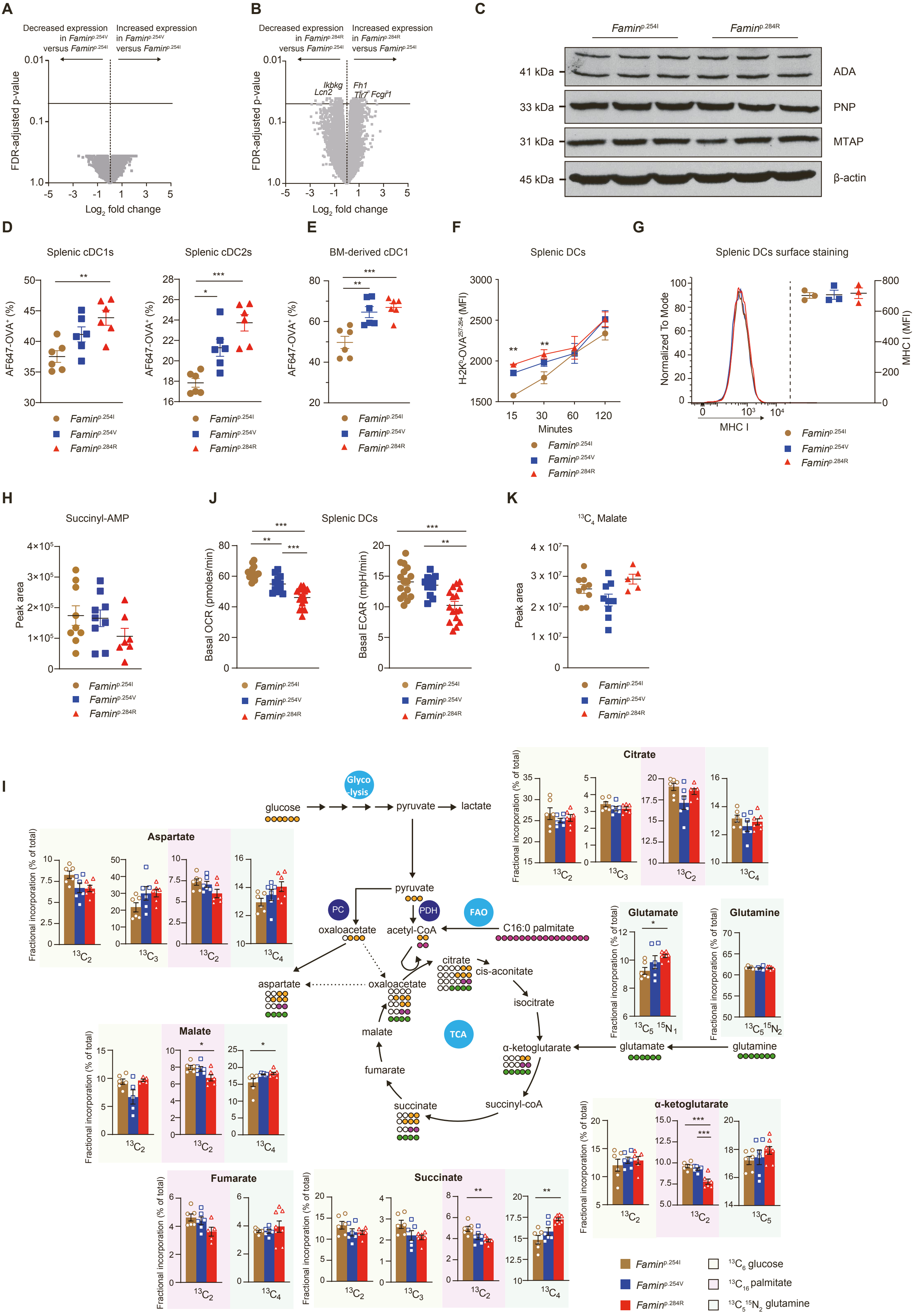

Figure S3. FAMIN activity in DCs biochemically controls antigen uptake and presentation. Related to Figure 3.

(A and B) Differentially expressed genes between *Famin*$^{p.254I}$ and *Famin*$^{p.254V}$ (A) and *Famin*$^{p.254I}$ and *Famin*$^{p.284R}$ (B) BM-derived cDC1s. Data depicted as volcano plot showing false discovery rate (FDR)-adjusted p value and $\log_2$ fold change (*n* = 5-6; GEO accession number GSE126473). List of differentially expressed genes for *Famin*$^{p.254I}$ versus *Famin*$^{p.284R}$ comparison is presented in Table S2.

(C) Immunoblots showing protein levels of ADA, PNP and MTAP in *Famin*$^{p.254I}$, *Famin*$^{p.284R}$ BM-derived cDC1s (*n* = 3).

(D) AF647-OVA$^+$ cells from *Famin*$^{p.254I}$, *Famin*$^{p.254V}$and *Famin*$^{p.284R}$ splenic DCs, gating on CD11c$^+$MHC II$^+$CD8$^+$CD11b$^-$ (cDC1s) or CD11c$^+$MHC II$^+$CD11b$^+$CD64$^-$ (cDC2s) (*n* = 6, 3 mice per genotype; please note control panel is shared with S4B).

(E) AF647-OVA$^+$ cells from *Famin*$^{p.254I}$, *Famin*$^{p.254V}$and *Famin*$^{p.284R}$ BM-derived cDC1s following incubation with AF647-OVA for 30 min (*n* = 6, 3 mice per genotype).

(F) MFI of *Famin*$^{p.254I}$, *Famin*$^{p.254V}$, *Famin*$^{p.284R}$ splenic DCs staining positive for 25-D1.16 mAb, which recognises the H-2K^b-OVA$^{257\text{-}264}$ complex, after incubation with 1 μg/ml OVA$^{257\text{-}264}$ for indicated times (*n* = 3).

(G) MHC I overlays and MFI of *Famin*$^{p.254I}$, *Famin*$^{p.254V}$, *Famin*$^{p.284R}$ splenic DCs stained with anti-MHC I antibody (*n* = 3).

(H) Succinyl-AMP levels in *Famin*$^{p.254I}$, *Famin*$^{p.254V}$and *Famin*$^{p.284R}$ BM-derived cDC1s (*n* = 7-9, 3 mice per genotype).

(I) Schematic and bar charts depicting metabolic fate of [$^{13}C_6$] stable isotope-labelled glucose after a 2 g/L pulse of *Famin*$^{p.254I}$, *Famin*$^{p.254V}$ and *Famin*$^{p.284R}$ BM-derived cDC1s for 1 h, and fate of 100 μM [$^{13}C_{16}$]-labelled palmitate and 2 mM [$^{15}N_2$ $^{13}C_5$]-

labelled glutamine following a 3 h pulse of *Famin*$^{p.254I}$, *Famin*$^{p.254V}$ and *Famin*$^{p.284R}$ BM-derived cDC1s. PC, pyruvate carboxylase; PDH, pyruvate dehydrogenase. Fraction of glutamine, glutamate, aspartate and TCA intermediates labelled as the indicated isotopomers is depicted in bar charts (*n* = 6-8, from 3-4 mice per genotype). Following a [$^{13}C_6$] glucose pulse, [$^{13}C_2$]-labelled TCA and aspartate isotopomers represent carbon flux from pyruvate which enters the TCA cycle via the PDH-catalysed generation of acetyl CoA, whereas the [$^{13}C_3$] isotopomers are derived from glucose anaplerosis via PC to generate oxaloacetate. [$^{13}C_2$] and [$^{13}C_3$]-labelled fumarate isotopomers were not detected following a 1 h pulse with [$^{13}C_6$]-labelled glucose.

(J) Basal oxygen consumption rate (OCR) and extracellular acidification rate (ECAR) of *Famin*$^{p.254I}$, *Famin*$^{p.254V}$ and *Famin*$^{p.284R}$ splenic DCs (*n* = 12-16 per group, 3 mice per genotype).

(K) Levels of cellular [$^{13}C_4$] malate following a 3 h pulse of *Famin*$^{p.254I}$, *Famin*$^{p.254V}$ and *Famin*$^{p.284R}$ BM-derived cDC1s with 300 µM [$^{13}C_4$] malate (*n* = 9/9/5, from 3/3/2 mice per genotype).

Data represented as mean ± S.E.M. *p < 0.05, **p < 0.01, and ***p < 0.001 (one-way ANOVA).

Figure S4. Adenine-guanine nucleotide interconversion controls antigen uptake and T cell priming. Related to Figure 4.

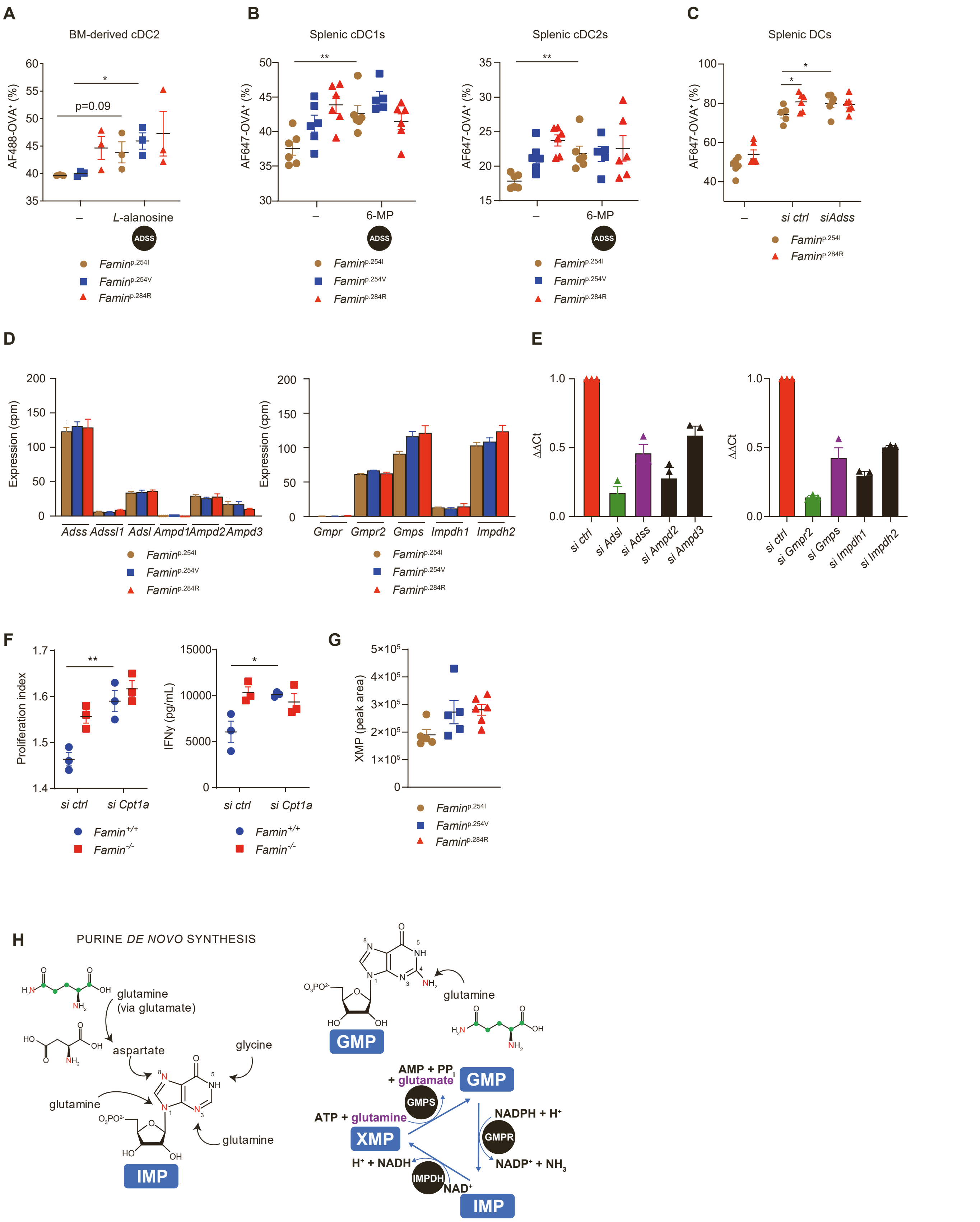

Figure S4. Adenine-guanine nucleotide interconversion controls antigen uptake and T cell priming by DCs. Related to Figure 4.

(A) Percentage AF488-OVA^{+} cells following incubation of *Famin*$^{p.254I}$, *Famin*$^{p.254V}$, *Famin*$^{p.284R}$ BM-derived cDC2s in presence of 25 μM *L*-alanosine (*n* = 3).

(B) Percentage AF647-OVA^{+} cells in *Famin*$^{p.254I}$, *Famin*$^{p.254V}$ or *Famin*$^{p.284R}$ CD11c^{+} DCs, gating on CD11c^{+}MHC II^{+}CD8^{+}CD11b^{-} (cDC1s) or CD11c^{+}MHC II^{+}CD11b^{+} CD64^{-} (cDC2s) cultured with vehicle, or in the presence of 5 μM 6-mercaptopurine (6-MP) (*n* = 6, from 3 mice per genotype; please note control panel is shared with S3D).

(C) Percentage AF647-OVA^{+} cells following incubation of *Famin*$^{p.254I}$ and *Famin*$^{p.284R}$ splenic CD11c^{+} DCs, 48 h after transfection with *ctrl* or *Adss* siRNA (*n* = 5/6, 3 mice per genotype, one-tailed unpaired t-test to compare *siCtrl* vs *siAdss*).

(D) Expression levels of *Adss, Adssl1, Adsl, Ampd1, Ampd2, Ampd3, Gmpr, Gmpr2, Gmps, Impdh1* and *Impdh2* in *Famin*$^{p.254I}$, *Famin*$^{p.254V}$, *Famin*$^{p.284R}$ cDC1s from RNA-Seq data (*n* = 5-6, GEO accession number GSE126473).

(E) $\Delta\Delta C_t$ depicted as fold change of *Adsl, Adss, Ampd2* and *Ampd3* or *Gmpr2, Gmps, Impdh1* and *Impdh2* expression relative to *si ctrl* (*n* = 3, representative of nucleofection experiments).

(F) Proliferation index and IFNγ released from OT-I T cells co-cultured for 72 h with 1 mg/ mL ovalbumin-pulsed *Famin*$^{+/+}$ and *Famin*$^{-/-}$ splenic DCs 48 h after nucleofection with *Cpt1a* or *ctrl* siRNAs (*n* = 3).

(G) XMP levels in *Famin*$^{p.254I}$, *Famin*$^{p.254V}$ and *Famin*$^{p.284R}$ cDC1s (*n* = 5/5/6, from 3 mice per genotype).

(H) IMP and GMP structural formulas highlighting the origin of the nitrogen atoms of the purine ring during *de novo* purine nucleotide synthesis and during GMPS-dependent transfer of the amide nitrogen from glutamine to XMP to form GMP.

Data represented as mean ± S.E.M. *p < 0.05, **p < 0.01, and ***p < 0.001 (one-way ANOVA or unpaired, two-tailed Student’s t test where appropriate).

Figure S5. Cytoplasmic NADH/ NAD⁺ redox state controls the pace of antigen uptake and MHC I recycling. Related to Figure 5.

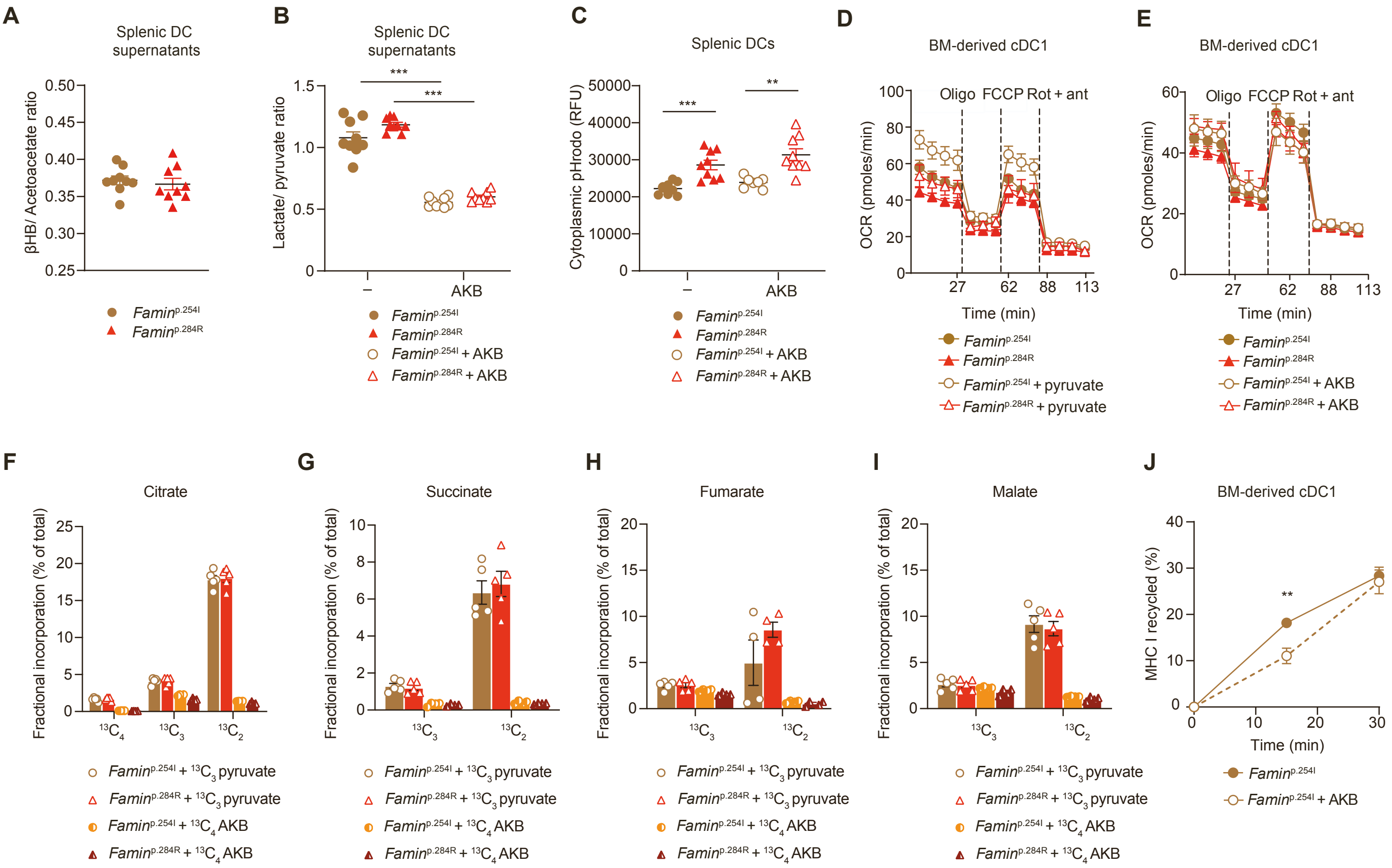

Figure S5. Cytoplasmic NADH/NAD$^+$ redox state controls the pace of antigen uptake and MHC I recycling. Related to Figure 5.

(A) Relative ratios of secreted β-hydroxybutyrate (β-HB) to acetoacetate, reflective of free mitochondrial NADH/NAD$^+$, in supernatants of *Famin*$^{p.254I}$ and *Famin*$^{p.284R}$ splenic DCs incubated overnight in RPMI-1640/10% FBS (*n* = 9, from 3 mice per genotype).
(B) Ratio of secreted lactate to pyruvate, reflecting free cytoplasmic NADH/NAD$^+$, in in supernatants of *Famin*$^{p.254I}$ and *Famin*$^{p.284R}$ splenic DCs incubated overnight in RPMI-1640/10% FBS in the presence or absence of 1 mM of α-ketobutyrate (AKB) (*n* = 9, from 3 mice per genotype).
(C) pH_c of *Famin*$^{p.254I}$ and *Famin*$^{p.284R}$ splenic DCs in the presence or absence of 1 mM of α-ketobutyrate (AKB) measured using pHrodo indicator probe (*n* = 7-9, from 3 mice per genotype).
(D and E) Oxygen consumption rate (OCR) of *Famin*$^{p.254I}$ and *Famin*$^{p.284R}$ BM-derived cDC1s in the presence or absence of 1 mM of pyruvate (D) or 1 mM of α-ketobutyrate (AKB) (E) (*n* = 6-14 per group, 3 mice per genotype).
(F-I) Fractional incorporation of [$^{13}C_3$] pyruvate or [$^{13}C_4$] α-ketobutyrate (AKB) into indicated isotopomers of the TCA intermediates citrate (F), succinate (G), fumarate (H) and malate (I) in *Famin*$^{p.254I}$ and *Famin*$^{p.284R}$ BM-derived cDC1s following a 3 h pulse of 1 mM [$^{13}C_3$] pyruvate or [$^{13}C_4$] α-ketobutyrate as indicated (*n* = 5/5/4/4 per group from 2/3 mice per genotype).
(J) Percentage of MHC I recycled in *Famin*$^{p.254I}$ BM-derived cDC1s at indicated times following overnight incubation with 1 mM AKB or control; AKB was replenished during the assay (*n* = 6, 3 mice per genotype).

Data represented as mean ± S.E.M. *$p < 0.05$, **$p < 0.01$, and ***$p < 0.001$ (one-way ANOVA or unpaired, two-tailed Student’s t test where appropriate).

Figure S6. FAMIN-dependent release of a soluble mediator by DCs controls T cell activation. Related to Figure 6.

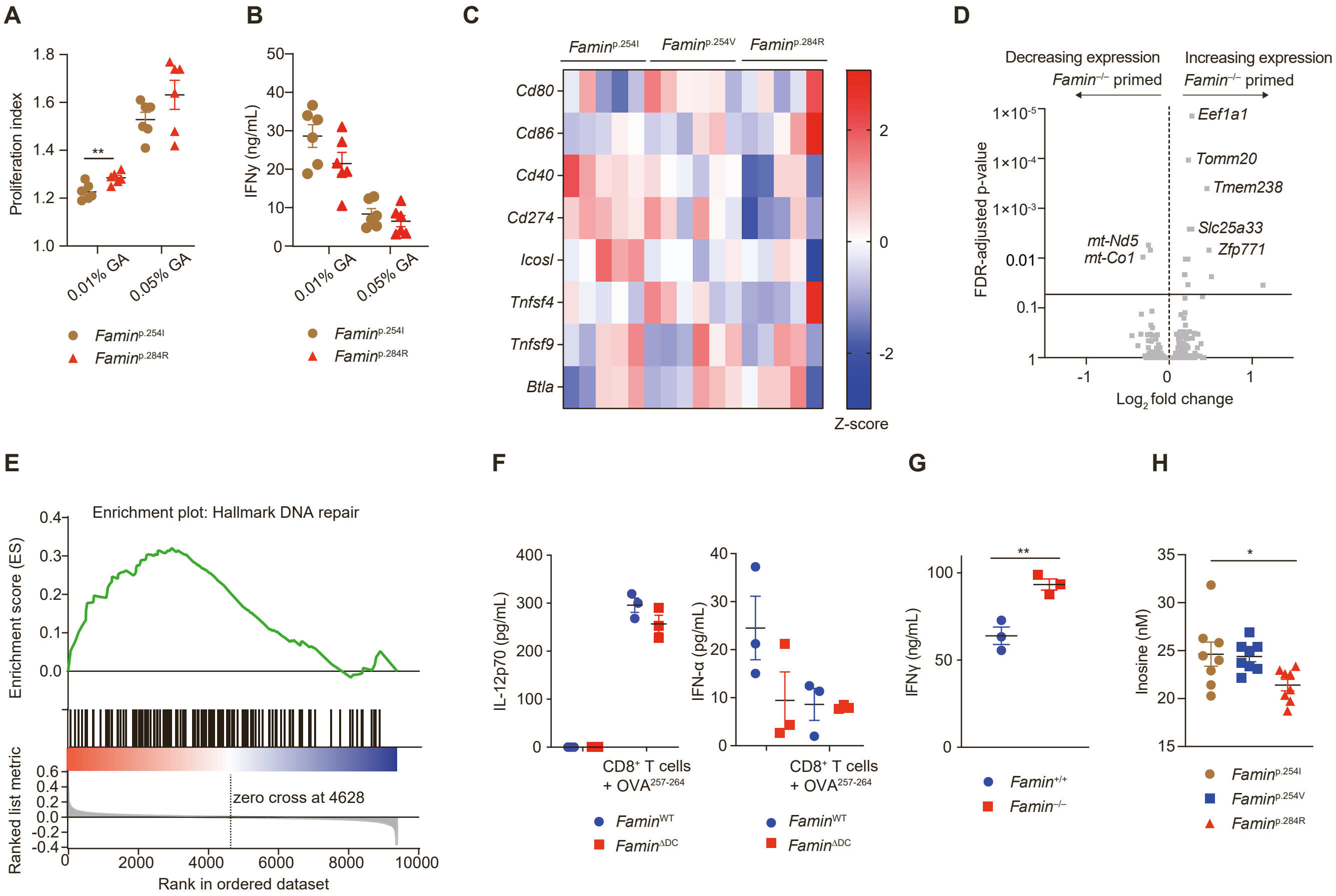

Figure S6. FAMIN-dependent release of a soluble mediator by DCs controls T cell activation. Related to Figure 6.

(A and B) Proliferation indices (A) and IFNγ release (B) from OT-I T cells cultured with 0.01% or 0.05% glutaraldehyde-fixed (GA) $OVA^{257\text{-}264}$-pulsed splenic $\textit{Famin}^{p.254I}$ or $\textit{Famin}^{p.284R}$ DCs (*n* = 6, from 3 mice per genotype).

(C) Heat map showing relative expression of co-stimulatory and co-inhibitory molecules in $\textit{Famin}^{p.254I}$, $\textit{Famin}^{p.254V}$ and $\textit{Famin}^{p.284R}$ BM-derived cDC1s, from RNA-Seq dataset (*n* = 5-6; GEO accession number GSE126473).

(D) Differentially expressed genes in anti-CD3/CD28 activated OT-I T cells cultured in the presence of 2 h supernatants of $\textit{Famin}^{-/-}$ or $\textit{Famin}^{+/+}$ splenic $CD11c^{+}$ DC. Data depicted as volcano plot showing p value (after Benjamini-Hochberg false discovery rate adjustment) and $\log_2$ fold change (*n* = 4 per group; GEO accession number GSE147370).

(E) Enrichment plots for the Hallmark gene set 'DNA repair' in the transcriptome of OT-I T cells cultured with $\textit{Famin}^{-/-}$ supernatant compared to T cells cultured with $\textit{Famin}^{+/+}$ supernatant (*n* = 4 per group).

(F) Secretion of IL-12p70 and IFN-α in supernatants from $\textit{Famin}^{WT}$ or $\textit{Famin}^{\Delta DC}$ splenic DCs pulsed with 1 μg/ml $OVA^{257\text{-}264}$ and cultured for 48 h with naïve OT-I T cells (*n* = 3).

(G) IFNγ levels from OT-I T cells activated by anti-CD3/CD28 in presence of supernatants from $\textit{Famin}^{+/+}$ or $\textit{Famin}^{-/-}$ splenic DCs cultured for 3 h in OptiMEM (*n* = 3).

(H) Absolute quantification of inosine in supernatants from $\textit{Famin}^{p.254I}$, $\textit{Famin}^{p.254V}$ and $\textit{Famin}^{p.284R}$ splenic DCs cultured in OptiMEM for 3 h (*n* = 8, 3 mice per genotype).

Data represented as mean ± S.E.M. *$p < 0.05$, **$p < 0.01$, and ***$p < 0.001$ (unpaired, two-tailed Student's t test where appropriate).

Figure S7. FAMIN-dependent conversion of extracellular hypoxanthine into inosine. Related to Figure 7.

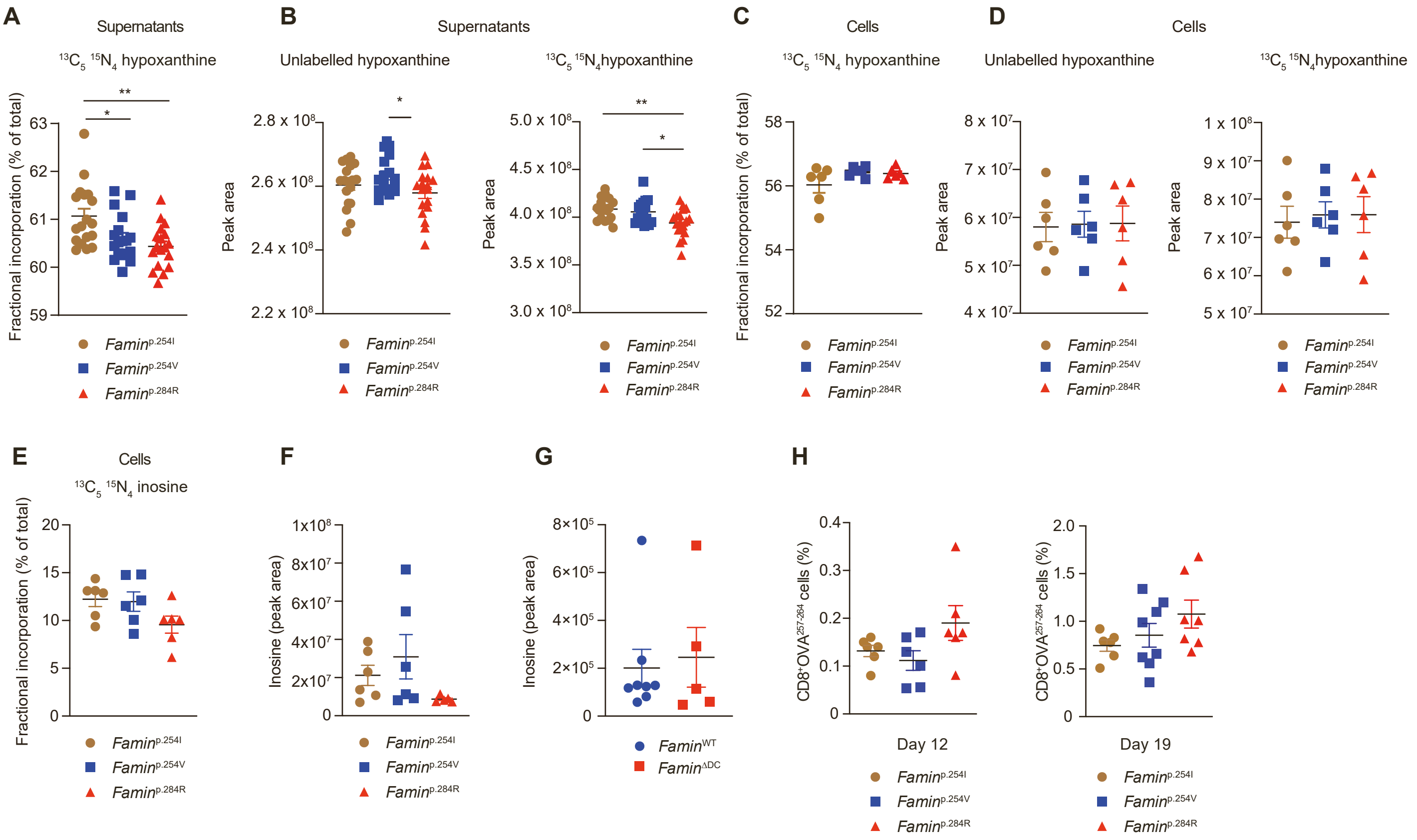

Figure S7. FAMIN-dependent conversion of extracellular hypoxanthine into inosine. Related to Figure 7.

(A-B) Fractional incorporation into [$^{13}C_5{}^{15}N_4$] hypoxanthine (as % of total hypoxanthine) (A), and absolute levels of unlabelled and labelled hypoxanthine (B) in supernatants of *Famin*$^{p.254I}$, *Famin*$^{p.254V}$ and *Famin*$^{p.284R}$ splenic DCs after a 3 h pulse with 25 μM [$^{13}C_5{}^{15}N_4$] hypoxanthine in OptiMEM (n = 18, 3 mice per genotype).

(C-D) Fractional incorporation into cellular [$^{13}C_5{}^{15}N_4$] hypoxanthine (C) and absolute levels of unlabelled and labelled hypoxanthine (D) in *Famin*$^{p.254I}$, *Famin*$^{p.254V}$, *Famin*$^{p.284R}$ splenic DCs following a 3 h pulse with [$^{13}C_5{}^{15}N_4$] hypoxanthine in OptiMEM (n = 6, 3 mice per genotype).

(E) Fractional incorporation into cellular [$^{13}C_5{}^{15}N_4$] inosine in *Famin*$^{p.254I}$, *Famin*$^{p.254V}$, *Famin*$^{p.284R}$ splenic DCs following a 3 h pulse with [$^{13}C_5{}^{15}N_4$] hypoxanthine in OptiMEM (n = 6, 3 mice per genotype).

(F) Inosine levels in plasma of *Famin*$^{p.254I}$, *Famin*$^{p.254V}$, *Famin*$^{p.284R}$ mice (n = 6/6/5).

(G) Inosine levels in plasma of *Famin*^{WT} or *Famin*$^{\Delta DC}$ mice (n = 8/5).

(H) % CD8$^+$ OVA$^{257\text{-}264}$ tetramer$^+$ CD3$^+$ CD45$^+$ cells from peripheral blood of *Famin*$^{p.254I}$, *Famin*$^{p.254V}$, *Famin*$^{p.284R}$ mice on day 12 and day 19 after inoculation with 2.5 × 10^4 LL2-OVA cells (n = 6/6/6 or 6/8/7).

Data represented as mean ± S.E.M. *$p < 0.05$, **$p < 0.01$, and ***$p < 0.001$ (unpaired, two-tailed Student's t test, one-way ANOVA or 2-way ANOVA where appropriate).

Table S1: CD8$^+$ and CD4$^+$ T cell numbers and splenic DC subsets in *Famin*$^{p.254I}$, *Famin*$^{p.254V}$ and *Famin*$^{p.284R}$ mice. Related to Figure 2.

	live CD3$^+$CD45$^+$	% cells			S.E.M.		
		Famin$^{p.254I}$	*Famin*$^{p.254V}$	*Famin*$^{p.284R}$	*Famin*$^{p.254I}$	*Famin*$^{p.254V}$	*Famin*$^{p.284R}$
CD8$^+$	Spleen	32.5	31.5	31.7	2.0	1.3	0.3
	MLN	39.3	38.2	37.4	1.0	0.6	0.8
CD4$^+$	Spleen	58.6	57.8	60.9	2.3	1.5	0.5
	MLN	56.7	57.6	55.3	2.3	2.9	1.4
		% cells			S.E.M.		
live CD11c$^+$MHC II$^+$		*Famin*$^{p.254I}$	*Famin*$^{p.254V}$	*Famin*$^{p.284R}$	*Famin*$^{p.254I}$	*Famin*$^{p.254V}$	*Famin*$^{p.284R}$
Spleen	cDC1	12.0	9.6	8.6	1.4	1.0	1.2
	cDC2	75.1	78.6	80.3	1.6	1.6	1.8

Table S1. $CD8^+$ and $CD4^+$ T cell numbers and splenic DC subsets in *Famin*$^{p.254I}$, *Famin*$^{p.254V}$ and *Famin*$^{p.284R}$ mice.

Table showing % of $CD8^+$ and $CD4^+$ T cells isolated from spleen or mesenteric lymph nodes (MLN) of *Famin*$^{p.254I}$, *Famin*$^{p.254V}$ and *Famin*$^{p.284R}$ mice, as a % of live $CD3^+CD45^+$ cells (n = 3), and % of cDC1s and cDC2s in spleens as a % total gated live $CD11c^+$MHC II^+ cells (n = 3). For gating strategy see Table S7. Data represented as mean ± S.E.M.

Table S2. Differentially expressed genes between *Famin*$^{p.254I}$ and *Famin*$^{p.284R}$ cDC1s from RNA-Seq analysis. Related to Figure S3B.

Gene	Log_2 fold change	P value	Adjusted p value	B
Gm5086	3.173	7.63E-06	3.14E-02	-0.471
Ociad2	2.185	1.73E-04	4.44E-02	0.094
Gins1	1.793	5.62E-06	3.14E-02	2.919
Fcgr1	1.620	2.51E-04	4.68E-02	0.264
Pclaf	1.483	1.01E-04	4.30E-02	1.451
Plac8	1.389	2.49E-05	4.10E-02	2.747
Birc5	1.310	3.64E-05	4.10E-02	2.319
Dut	1.310	2.67E-05	4.10E-02	2.664
Lipg	1.288	1.13E-04	4.35E-02	1.221
Enpp4	1.269	2.65E-05	4.10E-02	2.589
Bex6	1.266	1.56E-04	4.44E-02	1.074
Ticrr	1.230	8.29E-05	4.10E-02	1.535
Pole	1.223	7.13E-05	4.10E-02	1.787
Fignl1	1.222	1.92E-04	4.45E-02	0.862
Ndc80	1.184	1.04E-04	4.31E-02	1.313
Tpx2	1.181	6.46E-06	3.14E-02	4.047
Bhlhb9	1.126	4.34E-05	4.10E-02	2.224
Cdk1	1.118	5.78E-05	4.10E-02	1.965
Ccnb1	1.088	1.34E-04	4.44E-02	1.208
Tipin	1.072	6.57E-05	4.10E-02	1.872
Mef2c	1.055	1.83E-04	4.44E-02	0.856
Tlr7	1.050	3.02E-04	4.74E-02	0.346
Prr11	1.048	1.88E-04	4.45E-02	0.885
Pola1	1.022	2.24E-04	4.61E-02	0.730
Ddx60	1.007	1.44E-04	4.44E-02	1.078
Cenpe	0.966	1.60E-04	4.44E-02	1.035
Top2a	0.922	3.42E-04	4.85E-02	0.204
Lgals9	0.839	7.89E-05	4.10E-02	1.630
E2f8	0.829	2.93E-04	4.74E-02	0.484
Atad2	0.813	3.11E-04	4.80E-02	0.328
Mcm6	0.803	3.15E-04	4.80E-02	0.288
Cbx3	0.779	1.63E-04	4.44E-02	0.963
Psat1	0.755	2.85E-04	4.74E-02	0.457
Rbm43	0.753	4.08E-05	4.10E-02	2.318
Cenpa	0.750	9.81E-05	4.30E-02	1.430
Trim59	0.740	3.18E-04	4.80E-02	0.400
NA	0.740	2.69E-04	4.68E-02	0.521
Fam111a	0.728	1.94E-04	4.45E-02	0.760

Mcm4	0.708	2.56E-04	4.68E-02	0.508
BC035044	0.700	4.11E-05	4.10E-02	2.272
Tbc1d4	0.677	1.47E-04	4.44E-02	1.022
Hmgb2	0.670	2.51E-04	4.68E-02	0.510
Paics	0.597	3.42E-04	4.85E-02	0.206
Nasp	0.593	3.41E-04	4.85E-02	0.245
Rbl1	0.555	2.55E-04	4.68E-02	0.522
Herc3	0.543	2.98E-04	4.74E-02	0.371
Rpl39	0.535	8.88E-05	4.22E-02	1.513
Fh1	0.531	2.26E-04	4.61E-02	0.616
Rps24	0.520	2.66E-04	4.68E-02	0.461
Cops3	0.508	5.68E-05	4.10E-02	1.975
Rpl36a	0.472	1.72E-04	4.44E-02	0.867
Rps27a	0.450	3.03E-04	4.74E-02	0.328
Anp32e	0.415	1.32E-04	4.44E-02	1.129
Rpl22	0.400	1.80E-04	4.44E-02	0.826
Eef1b2	0.389	2.35E-04	4.68E-02	0.568
Rpl27a	0.345	2.17E-04	4.61E-02	0.646
Brd4	-0.365	1.48E-04	4.44E-02	1.016
Ikbkg	-0.392	2.23E-04	4.61E-02	0.617
Clcn7	-0.489	1.57E-04	4.44E-02	0.965
Sec14l1	-0.492	3.45E-04	4.85E-02	0.213
Zfp142	-0.509	2.04E-04	4.58E-02	0.792
Rabgef1	-0.510	6.64E-05	4.10E-02	1.839
Flot2	-0.512	3.22E-04	4.80E-02	0.390
Ralgds	-0.520	9.92E-05	4.30E-02	1.476
Zdhhc18	-0.540	7.74E-05	4.10E-02	1.663
Mir22hg	-0.589	2.20E-04	4.61E-02	0.644
Washc1	-0.608	1.16E-04	4.35E-02	1.299
Plxna1	-0.646	6.87E-05	4.10E-02	1.761
Mllt6	-0.647	6.12E-05	4.10E-02	1.881
Fhl3	-0.669	2.27E-04	4.61E-02	0.643
Plekhm2	-0.683	1.77E-04	4.44E-02	0.860
Sufu	-0.712	2.59E-04	4.68E-02	0.587
Abcc1	-0.727	4.42E-05	4.10E-02	2.192
Pgd	-0.785	2.77E-04	4.74E-02	0.409
Mafg	-0.858	4.54E-05	4.10E-02	2.211
Scrib	-0.867	3.32E-04	4.85E-02	0.350
Arrb1	-0.916	1.24E-04	4.40E-02	1.183
Smox	-0.926	8.06E-05	4.10E-02	1.659
Mier2	-0.975	2.93E-04	4.74E-02	0.460
Mylip	-0.983	2.49E-04	4.68E-02	0.600
Abcc3	-1.205	1.79E-04	4.44E-02	0.882

Lcn2	-1.262	1.20E-04	4.38E-02	1.216
Slc16a3	-1.308	2.65E-04	4.68E-02	0.579
Exoc3l4	-1.381	7.53E-05	4.10E-02	1.728
Arap3	-1.632	5.52E-05	4.10E-02	1.243
I830127L07Rik	-1.637	1.12E-04	4.35E-02	1.188
Kcnh3	-1.931	3.03E-04	4.74E-02	-0.557
Rps4l	-2.567	1.48E-04	4.44E-02	-1.387

Table S2. Differentially expressed genes between *Famin*$^{p.254I}$ and *Famin*$^{p.284R}$ cDC1s from RNA-Seq analysis. Related to Figure S3B.

Table showing differentially expressed genes between *Famin*$^{p.254I}$ and *Famin*$^{p.284R}$ bone marrow-derived cDC1s, with corresponding $\log_2$ fold changes and FDR-adjusted p values (n = 5; GEO accession number GSE126473).

Table S3: Comparison of purine nucleotide levels in *Famin*$^{p.254I}$, *Famin*$^{p.254V}$ and *Famin*$^{p.284R}$ bone marrow-derived cDC1s. Related to Figure 3B.

	**Normalised peak area (mean)**			**S.E.M.**			**Fold change compared to *Famin*$^{p.254I}$**	
	***Famin*$^{p.254I}$**	***Famin*$^{p.254V}$**	***Famin*$^{p.284R}$**	***Famin*$^{p.254I}$**	***Famin*$^{p.254V}$**	***Famin*$^{p.284R}$**	***Famin*$^{p.254V}$**	***Famin*$^{p.284R}$**
ATP	2471023	1368797	609597	633381	324166	71344	0.55	0.25
ADP	6897799	3612773	1833730	1252405	792128	103916	0.52	0.27
AMP	16524345	11894852	6564931	3587605	2760995	1170307	0.72	0.40
GTP	271433	143922	61311	81172	42741	8545	0.53	0.23
GDP	715115	369813	200879	115422	81920	21810	0.52	0.28
GMP	908715	613638	372708	101696	134098	64498	0.68	0.41
IMP	169291	126088	85074	15853	14545	8705	0.74	0.50

Table S3. Comparison of purine nucleotide levels in *Famin*$^{p.254I}$, *Famin*$^{p.254V}$ and *Famin*$^{p.284R}$ bone marrow-derived cDC1s. Related to Figure 3B.

Table showing levels of indicated nucleotides in bone marrow-derived *Famin*$^{p.254I}$, *Famin*$^{p.254V}$ and *Famin*$^{p.284R}$ cDC1s, presented as peak areas normalised to total ion content ± S.E.M. Corresponding fold changes of nucleotide levels in *Famin*$^{p.254V}$ and *Famin*$^{p.284R}$ cDC1s compared to *Famin*$^{p.254I}$ cDC1s are also indicated.

Table S4: Co-inhibitory and co-stimulatory molecules expressed in *Famin*$^{p.254I}$, *Famin*$^{p.254V}$ and *Famin*$^{p.284R}$ DCs. Related to Figure S6C.

					S.E.M.			
	***Famin*$^{+/+}$**	***Famin*$^{–/–}$**	***Famin*$^{+/+}$**	***Famin*$^{–/–}$**	***Famin*$^{+/+}$**	***Famin*$^{–/–}$**	***Famin*$^{+/+}$**	***Famin*$^{–/–}$**
	**DCs**		**DCs + T cells**		**DCs**		**DCs + T cells**	
CD86 (%)	21.6	21.6	39.9	42.6	1.0	0.5	1.0	3.6
CD80 (%)	27.1	27.3	50.1	42.4	1.3	1.2	1.5	2.5
CD40 (%)	4.3	5.6	14.0	12.9	0.2	0.7	0.8	0.2
ICOSL (%)	35.4	35.6	16.1	15.0	1.6	5.3	1.6	2.2
PD-L1 (%)	34.3	28.3	58.0	55.8	1.4	0.8	1.4	2.5
MHC I (MFI)	5333.0	4916.0	4186.7	4085.3	259.7	661.8	84.4	204.5
OX40-L (MFI)	393.7	399.7	112.7	96.4	17.5	52.7	14.2	4.5
MHC II (MFI)	1710.3	1618.3	1764.3	1703.7	25.2	170.0	73.0	119.2

Table S4: Co-inhibitory and co-stimulatory molecules expressed in *Famin*$^{p.254I}$, *Famin*$^{p.254V}$ and *Famin*$^{p.284R}$ DCs. Related to Figure S6C.

Mean fluorescence intensity of co-stimulatory and co-inhibitory molecules of *Famin*$^{+/+}$ and *Famin*$^{–/–}$ CD11c^{+} splenic DCs cultured alone or in co-culture with OT-I CD8^{+} T cells for 24 h after a pulse with 1 μg/mL of OVA$^{257-264}$ (*n* = 3).

Table S5: Nucleotide changes in *Famin*$^{p.254I}$ BM-derived cDC1s on incubation with Cpd3. Related to Figure 4G.

	Control	+Cpd3	Control	+Cpd3	
**Metabolite**	**Normalised peak area**		**S.E.M.**		**Fold change in Cpd3-treated versus control**
AMP	44199672	20249820	3230475	2142543	0.46
ADP	29384998	22824682	2406342	1663796	0.78
ATP	14477785	19486398	1725677	3437110	1.35
GMP	4673911	2623467	390093	359483	0.56
GDP	3269750	3243153	310368	239226	0.99
GTP	1883139	2610605	209515	356142	1.39
XMP	95912	286096	12349	33486	2.98
IMP	2972109	18007984	245197	2749628	6.06
Succinyl-AMP	88087	285601	10744	37273	3.24

Table S5: Nucleotide changes in *Famin*$^{p.254I}$ BM-derived cDC1s on incubation with Cpd3. Related to Figure 4G.

Table showing changes in purine nucleotide levels in bone marrow-derived *Famin*$^{p.254I}$ cDC1s upon incubation with 5 μM of Cpd3 for 18 h, compared with vehicle control. Data are presented as peak areas normalised to total ion content ± S.E.M. Fold changes in the Cpd3-treated samples compared to the vehicle control samples are also indicated.

Table S6: Gene set enrichment analysis of OT-I T cell RNA-Seq dataset. Related to Figures 6B-C and S6E.

Top 10 enriched Hallmark gene sets	Enrichment score	FDR q-value
OXIDATIVE PHOSPHORYLATION	1.56	0.08
DNA REPAIR	1.53	0.08
MYC TARGETS V2	1.59	0.12
UNFOLDED PROTEIN RESPONSE	1.30	0.41
CHOLESTEROL HOMEOSTASIS	1.31	0.46
FATTY ACID METABOLISM	1.14	0.69
REACTIVE OXYGEN SPECIES PATHWAY	1.18	0.76
MYC TARGETS V1	1.15	0.76
P53 PATHWAY	1.08	0.85
BILE ACID METABOLISM	1.05	0.86

Table S6. Gene set enrichment analysis of OT-I T cell RNA-Seq dataset. Related to Figure 6B-C and S6E.

Gene set enrichment analysis of RNA-Seq dataset comparing OT-I T cells cultured with *Famin*$^{-/-}$ supernatant compared to T cells cultured with *Famin*$^{+/+}$ supernatant (*n* = 4 per group; GEO accession number GSE147370). The top ten enriched Hallmark gene sets in order of FDR q value are shown.

Table S7. Gating strategies for flow cytometry panels. Related to STAR methods.

Gating strategy	Figure panels
$Sytox^{-}CD3^{+}CD4^{-}CD8^{+}(NP^{366\text{-}374+}/PA^{224\text{-}233+})$	Figures 1D, 1E; S1B
$Sytox^{-}CFSE^{+}$ (see Methods for calculation)	Figures 2D, 2H; S2A
$Sytox^{-}CD11c^{+}MHC\ II^{+}OVA647^{+}$	Figures 3D, 4A-D, 4H-I, 5A, 5H-I; S3E, S4C
$Sytox^{-}CD11c^{+}MHC\ II^{+}\ OVA488^{+}$	Figure S4A
$Sytox^{-}CD11c^{+}MHC\ II^{+}H\text{-}2K^{b}\text{-}OVA^{257\text{-}264+}$	Figures 3F; S3F
$Sytox^{-}CD3^{+}CD8^{+}CFSE^{+}$	Figures 2A-C, 4E, 4N-P, 6E, 6M-N, 6Q; S2B, S4F, S6A
$Sytox^{-}CD3^{+}CD8^{+}T\alpha2^{+}T\beta5.1^{+}CFSE^{+}$	Figure S2C
$Sytox^{-}CD3^{+}CD4^{+}CFSE^{+}$	Figure 2I
$FVD^{-}CD3^{+}CD4^{+}(IFN\gamma^{+}/IL\text{-}4^{+}/IL\text{-}17^{+}/Foxp3^{+})$	Figures 2L; S2D
$Sytox^{-}CD4^{+}CD45.1^{+}CFSE^{+}$	Figure 2M
$Sytox^{-}CD11c^{+}MHC\ II^{+}MHC\ I^{+}$	Figure S3G
$Sytox^{-}CD45^{-}CD3^{+}CD8^{+}OVA^{257\text{-}264+}$	Figure S7H
$Sytox^{-}CD45^{+}CD3^{+}CD8^{+}$ or $Sytox^{-}CD45^{+}CD3^{+}CD4^{+}$	Table S1
$Sytox^{-}CD11c^{+}MHC\ II^{+}$ ($CD11b^{+}CD64^{-}$ for cDC2 and $CD8^{+}CD11b^{-}$ for cDC1)	Table S1
$Sytox^{-}CD11c^{+}MHC\ II^{+}\ OVA647^{+}$ ($CD11b^{+}CD64^{-}$ for cDC2 and $CD8^{+}CD11b^{-}$ for cDC1)	Figures S3D, S4B

Table S7. Gating strategies for flow cytometry panels. Related to STAR methods.

Cell surface and intracellular markers used to characterise populations in corresponding panels of the figures. For detailed information on antibodies please see STAR Methods. Fixable viability dye is abbreviated as FVD.
